# The Effect of Electroencephalogram (EEG) Reference Choice on Information-Theoretic Measures of the Complexity and Integration of EEG Signals

**DOI:** 10.3389/fnins.2017.00425

**Published:** 2017-07-25

**Authors:** Logan T. Trujillo, Candice T. Stanfield, Ruben D. Vela

**Affiliations:** Department of Psychology, Texas State University San Marcos, TX, United States

**Keywords:** electroencephalography, EEG complexity, EEG integration, EEG spectral power density, EEG reference, resting state EEG

## Abstract

Converging evidence suggests that human cognition and behavior emerge from functional brain networks interacting on local and global scales. We investigated two information-theoretic measures of functional brain segregation and integration—interaction complexity C_*I*_(X), and integration I(X)—as applied to electroencephalographic (EEG) signals and how these measures are affected by choice of EEG reference. C_I_(X) is a statistical measure of the system entropy accounted for by interactions among its elements, whereas I(X) indexes the overall deviation from statistical independence of the individual elements of a system. We recorded 72 channels of scalp EEG from human participants who sat in a wakeful resting state (interleaved counterbalanced eyes-open and eyes-closed blocks). C_I_(X) and I(X) of the EEG signals were computed using four different EEG references: linked-mastoids (LM) reference, average (AVG) reference, a Laplacian (LAP) “reference-free” transformation, and an infinity (INF) reference estimated via the Reference Electrode Standardization Technique (REST). Fourier-based power spectral density (PSD), a standard measure of resting state activity, was computed for comparison and as a check of data integrity and quality. We also performed dipole source modeling in order to assess the accuracy of neural source C_I_(X) and I(X) estimates obtained from scalp-level EEG signals. C_I_(X) was largest for the LAP transformation, smallest for the LM reference, and at intermediate values for the AVG and INF references. I(X) was smallest for the LAP transformation, largest for the LM reference, and at intermediate values for the AVG and INF references. Furthermore, across all references, C_I_(X) and I(X) reliably distinguished between resting-state conditions (larger values for eyes-open vs. eyes-closed). These findings occurred in the context of the overall expected pattern of resting state PSD. Dipole modeling showed that simulated scalp EEG-level C_I_(X) and I(X) reflected changes in underlying neural source dependencies, but only for higher levels of integration and with highest accuracy for the LAP transformation. Our observations suggest that the Laplacian-transformation should be preferred for the computation of scalp-level C_I_(X) and I(X) due to its positive impact on EEG signal quality and statistics, reduction of volume-conduction, and the higher accuracy this provides when estimating scalp-level EEG complexity and integration.

## Introduction

**Figure 1:**
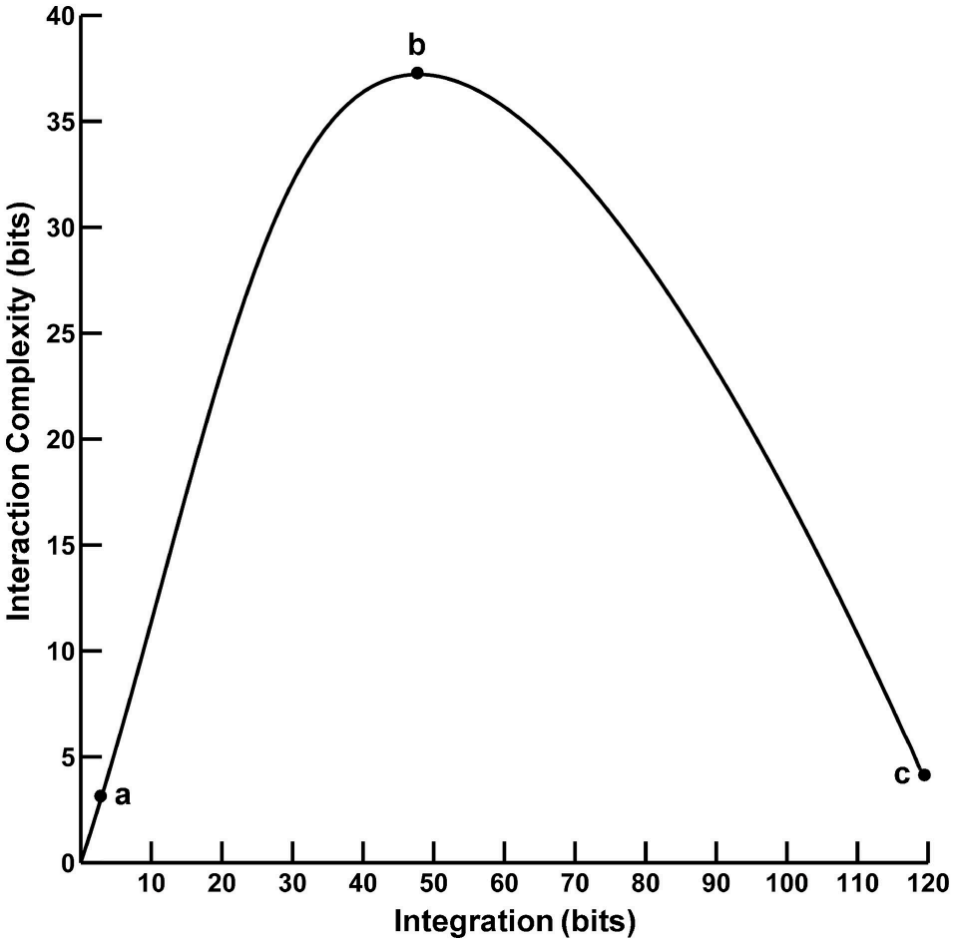
The functional relationship between interaction complexity C_I_(X) and integration I(X). In this example, interaction complexity C_I_(X) and integration I(X) were computed from constant mean Gaussian Toeplitz covariance matrices (*n* = 72) with increasing σ and 10% uncorrelated Gaussian noise added to the matrix diagonal (following Tononi et al., 1994). For low values of I(X) (case a), C_I_(X) is also low and all system components are fully statistically independent. For intermediate values of I(X) (case b), C_I_(X) is high; some system components are statistically dependent, others are not. For high values of I(X) (case c), C_I_(X) is low; the system components are fully dependent.

Converging evidence suggests that human cognition and behavior emerge from brain networks interacting on local and global scales. These different scales of neural activity reflect the functional *segregation* (specialized information processing within regional groups of brain regions) and *integration* (the combination of that specialized information across distributed brain regions) of the brain networks (Tononi et al., 1994, 1996, 1998a,b; Bullmore and Sporns, 2009; Fair et al., 2009; Rubinov and Sporns, 2010). Moreover, the organization of these brain networks is highly complex due to the dynamic interplay of segregation and integration. This *neural complexity* reflects a large number of coordinated interactions among brain elements engaged in various levels of subordination that are neither fully regular nor random (Tononi et al., 1998a). One approach to quantifying segregation, integration, and complexity in the brain utilizes information theory to characterize neural activity in terms of “deviations from statistical independence among components of a neural system” (Tononi et al., 1994, p. 5033). In this paper, we consider two such information-theoretic measures as applied to the analysis of electroencephalographic (EEG) data. The first measure, called *integration I(X)*, is a multivariate index of the overall deviation from statistical independence of the individual elements in a system. The second measure, called *interaction complexity C*_I_*(X)*, is a statistical measure of a system's information content that results from the interactions among the system's elements. The relationship between complexity and integration follows an “inverted-U” non-monotonic function (Tononi et al., 1994; see Figure 1). Complexity is low at low integration values when system components are fully statistically independent; complexity is high at intermediate integration values when there is heterogenous statistical dependence among system components (i.e., when a system is highly integrated and specialized; Tononi et al., 1998a), and complexity is low at high integration values when system components are fully statistically dependent. These measures are, in part, the precursors to the segregation and integration measures used in the current integrated information theory of consciousness and brain function (Tononi, 2004; Tononi and Koch, 2016).

In order for C_I_(X) and I(X) to provide useful insight into the brain networks underlying cognition and behavior, it is important to understand how they are influenced by the various parameters of EEG measurements. This allows one to assess the reliability and validity of C_I_(X) and I(X) under different measurement scenarios. One crucial EEG measurement parameter is the reference scheme or montage. The EEG signal represents a difference between two voltages, one located at an electrode site of interest and another at a location that is as electrically neutral as possible with respect to the signal of interest. The choice of EEG reference is well-known to affect signal quality (Gencer et al., 1996), as different reference choices may or may not be electrically neutral depending on location, participant behavior, and the neurocognitive process under investigation (Wolpaw and Wood, 1982; Desmedt et al., 1990; Dien, 1998; Yao, 2001; Trujillo et al., 2005). The impact of EEG reference on signal quality should also affect the reliability of the signal statistics from which these C_I_(X) and I(X) measures are derived. Furthermore, C_I_(X) and I(X) index the interaction between different brain signal elements, interdependencies that can be artificially-inflated at the level of the scalp due to the effects of volume conduction of cortical EEG source signals throughout the head (Nunez et al., 1997, 1999; Nunez and Srinivasan, 2006). Scalp EEG measurements made with respect to different reference schemes may be affected by volume conduction to different degrees, and this in turn should affect the degree to which these complexity measures reflect true or artifactual complexity and integration.

Thus, if such complexity and integration measures are to be useful when applied to scalp-recorded EEG data, it is first necessary to ascertain the reliability and stability of these measures with respect to different EEG references. Unfortunately, such studies are lacking in the current literature. To our knowledge, only one previous study (van Putten and Stam, 2001) has compared the effect of EEG reference on information-theoretic measures of integration and complexity. Van Putten and Stam applied I(X) and another EEG complexity measure called neural complexity C_N_(X) (which is related, but not identical, to interaction complexity) to the analysis of scalp-recorded EEG signals collected during a resting state task (eyes-closed and eyes-open condition). In this study, the EEG data were referenced to an average reference and a source reference (computed as the voltage difference between the recording site and the mean voltage of 3–4 neighboring recording sites) that served to reduce idiosyncratic reference effects and effects of volume conduction. The magnitude of I(X) and C_N_(X) were lower for the source reference compared to the average reference, although the overall between-condition pattern was the same for both references (greater I(X) and C_I_(X) for eyes closed than eyes open).

The goal of the present study was to perform a more comprehensive investigation into the effect of EEG reference on the quantification of EEG integration and complexity. We recorded 72 channels of scalp EEG from human participants who sat in a wakeful resting state (interleaved counterbalanced eyes-open and eyes-closed blocks). We then computed C_I_(X) and I(X) of the EEG signals, as well as a conventional EEG measure of resting state activity—Fourier-based power spectral density (PSD)—for comparison and as a check of data integrity and quality. We computed the information-theoretic and spectral power measures using four different EEG reference schemes. The first was the linked-mastoids (LM) reference, which consists of the mathematical average of the signals from electrodes located at the mastoid bones of each ear. The second scheme was the average (AVG) reference (Bertrand et al., 1985), computed by averaging the signals from all electrodes and then subtracting the averaged signal from each electrode individually. The third scheme was a Laplacian (LAP) “reference-free” transformation of the raw EEG potentials into a measure of the radial current density at the scalp (Law et al., 1993; Yao, 2002). Finally, the fourth reference scheme was an infinity (INF) reference, which uses the Reference Electrode Standardization Technique (REST; Yao, 2001) to approximately transform a scalp point reference (or the average reference) to a reference point at infinity. REST achieves this by computing the actual or equivalent neural sources for a set of EEG signals and then implementing a forward computation of the obtained sources to a point at infinity.

In order to assist interpretation of the observed EEG complexity and integration, we simulated oscillatory resting state EEG data at the scalp via a concentric 4-shell spherical head forward volume-conduction model (Cuffin and Cohen, 1979; Mosher et al., 1993; Tenke and Kayser, 2015). These simulations were based on oscillating, fixed-location intracranial dipole sources with pre-determined complexity and integration. This allowed us to investigate how accurately EEG source complexity and integration patterns could be estimated at the scalp, given the known mixing effects of volume conduction (Nunez and Srinivasan, 2006). We addressed four specific questions in this study: (1) what, if any, difference does choice of EEG reference make for empirical assessments of C_I_(X) and I(X); (2) how accurately does scalp EEG recordings reflect the complexity and integration of underlying sources; (3) can these measures reliably distinguish between neurocognitive conditions in which, in theory, complexity and integration should be different; and (4) does this between-condition sensitivity vary according to EEG reference scheme?

## Materials and methods

### Participants

Twenty two Texas State University undergraduates (11 female, 11 male, mean age = 21.1 ± 0.52 years, age range = 18—26) participated in this study for course credit or monetary payment. This study was carried out in accordance with the recommendations of the Human Subjects Institutional Review Board (IRB) at Texas State University with written informed consent from all participants. All participants gave written informed consent in accordance with the Declaration of Helsinki. The protocol was approved by the Texas State University IRB.

### Stimuli and procedure

After consent, participants underwent setup for EEG recording. During the setup, participants completed several questionnaires indexing demographic and health information, sleep quality and quantity, emotion and mood states, and current attentional states. However, the results of these questionnaires are not relevant to the topic of this paper and are not reported here.

#### Resting state EEG

After completion of the EEG setup, participants underwent 8 min of resting state EEG recording while sitting quietly in a comfortable padded chair in a darkened room (4 min eyes open and 4 min eyes closed interleaved in 1-min intervals; eyes open/closed order was balanced across participants). (Due to a technical recording error, one participant only received 4 min of recording time.) Subjects were instructed to remain relaxed, yet alert and awake at all times during recording. After completion of the resting state EEG recording, participants then performed a visual categorization task, the results of which are not relevant to the topic of this paper and thus are not reported here.

### EEG recording and pre-processing

**Figure 2:**
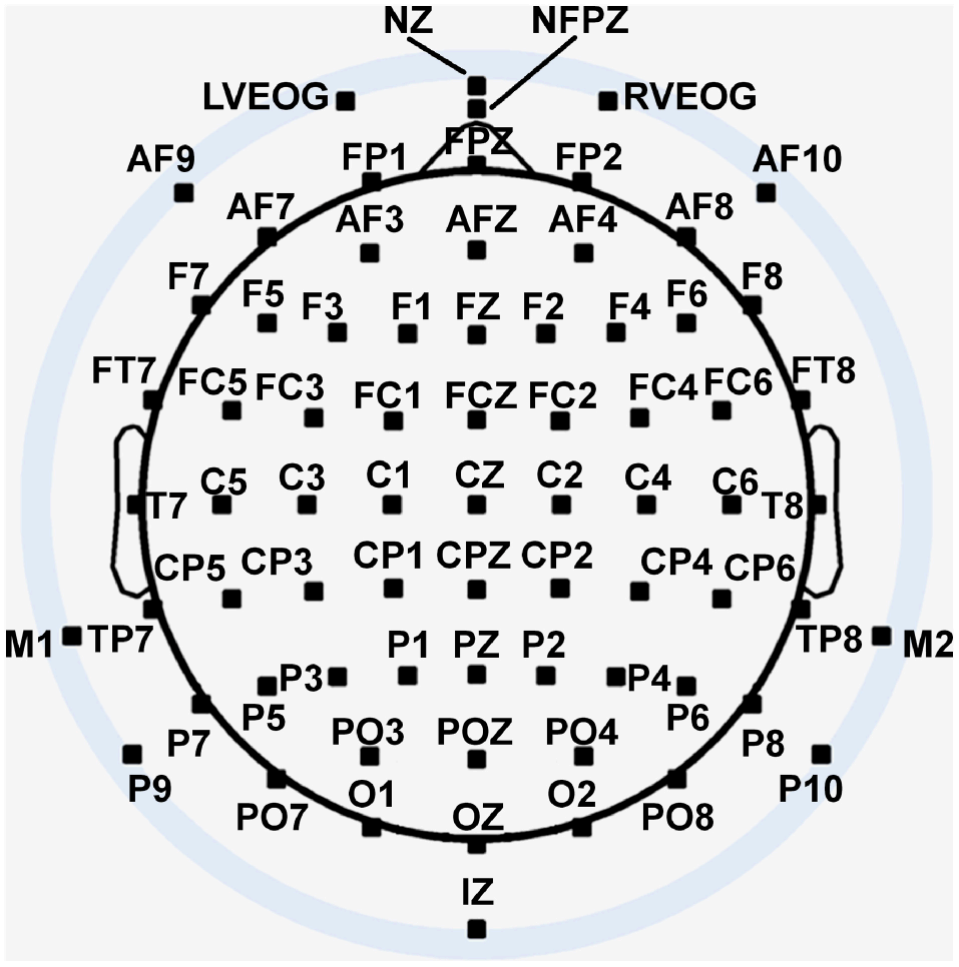
Extended 10–20 scalp locations of EEG recording electrodes. Note that sites outside the radius of the head represent locations that are below the equatorial plane (FPZ-T7-T8-OZ plane) of the (assumed spherical) head model. Sites LVEOG and HEOG were located below the eyes approximately at the same longitude as sites M1 and M2.

We recorded 72 channels of continuous EEG signals using active Ag/AgCl electrodes either mounted in a BioSemi electrode cap or via freestanding electrodes. Recording sites included international 10/5 system locations (Jurcak et al., 2007) and the inferior orbits of the eyes (Figure 2). All channels were amplified by a BioSemi Active II amplifier system in 24-bit DC mode at an initial sampling rate of 2,048 Hz (400-Hz bandwidth) downsampled online to 256 Hz, with EEG signals recorded with respect to a common mode sense (CMS) electrode located between sites PO3 and POZ. Half-cell potentials of the electrode/gel/skin interface were kept between ± 40 mV, following standard recommendations for the Active II system. EEG data were imported offline into the MATLAB computing software environment (The Math Works, Inc., Natick, MA, USA) using the EEGLAB toolbox (Delorme and Makeig, 2004) for MATLAB, where all subsequent analysis was performed via in-house scripts that utilized EEGLAB functions.

Resting EEG baseline data were divided into 1 s (256 sample) epochs with 50% overlap, initially producing 480 epochs for each of the two resting task conditions. Next, we created a copy of the resting EEG trials that were transformed to an average reference for the purpose of identifying bad channels and muscle and signal artifacts from the EEG record by visual inspection. Once identified, we then removed artifact-contaminated trials from, and replaced bad channels in, the original non-average-referenced EEG trials. Bad EEG channels were replaced using an EEGLAB-based spherical spline interpolation algorithm (Perrin et al., 1987; *m* = 50, 50 term expansion). No more than 3.5% of channels on average were interpolated for any given subject.

Blink and saccade-related electroocular (EOG) artifacts were removed by first computing two EOG channels: one formed from the bipolar montage of site NZ and the average of the two electrodes located at the inferior orbits of the eyes (sensitive to blinks and vertical saccades) and a second formed from the bipolar montage of AF9 and AF10 (sensitive to horizontal saccades). Next, EEG trials containing EOG amplitudes higher than 50 μV or lower than −50 μV (after removal of the constant direct current offset from the EOG signals) were rejected from the analysis in MATLAB via automatic algorithm. These rejection criteria were applied over the full epoch interval for resting EEG data. Then, a second round of manual artifact scoring was performed because the ocular artifact rejection algorithm occasionally failed to identify trials with ocular artifacts. The derived horizontal and vertical EOG channels were removed from the data after elimination of the EOG artifact-contaminated trials.

On average 314 ± 16 and 299 ± 20 trials remained for the eyes closed and open resting state conditions after artifact rejection. However, in order to avoid any potential between-condition differences in information bias (Pola et al., 2003; Misra et al., 2005; Magri et al., 2009; Ince et al., 2017) that might arise during the computation of the complexity and integration measures (see Computation of EEG Complexity and Integration section, below), the number of EEG trials were matched between resting state conditions. For each participant, we randomly sampled trials (without replacement) from the condition with the larger number of trials to match the smaller number of trials for the other condition. Thus, the final number of trials entering into each resting state condition was 270 ± 18 on average. Trials were also matched in this manner for computation of EEG spectral power for a cleaner comparison with the EEG complexity and integration analyses.

EEG trials were then converted into four reference montages examined in this study: linked-mastoids (LM) reference, average (AVG) reference, Laplacian (LAP) “reference free” transformation, and an infinity (INF) reference estimated via the Reference Electrode Standardization Technique (REST). LM and AVG references were created via standard derivations (Yao et al., 2007). The LAP transformation (μV/m^2^ units; unit sphere; 50 iterations; *m* = 4; λ = 10^−5^) was achieved using a spherical spline algorithm (Perrin et al., 1989, 1990) implemented in the CSD Toolbox for MATLAB (Kayser and Tenke, 2006a,b; Kayser, 2009; http://psychophysiology.cpmc.columbia.edu/Software/CSDtoolbox). The INF was estimated using the REST software for MATLAB (Tian and Yao, 2013; www.neuro.uestc.edu.cn/rest). All 72 electrodes were entered into the computation of the LAP transformation and the average- and infinite-references.

### Computation of resting EEG power spectral density

Resting EEG power spectral density (PSD; μV^2^/Hz) was computed on unfiltered data via Fast Fourier Transformation (FFT) tapered by a 1 s Hamming window (Kornguth et al., 2013; Witkowski et al., 2015). For each subject and resting state condition, mean PSD was computed and then converted into decibels (dB). The latter conversion allowed for direct comparison between the PSD computed for the LAP-transformed data (in units of μV^2^/m^4^/Hz), and the PSD computed for the data referenced to the other EEG references (in units of μV^2^/Hz). Additionally, the logarithmic transformation of the data during the process of conversion to dB units served to transform the PSD values toward a Gaussian statistical distribution, thus allowing application of parametric statistical tests (see Statistical Analysis of EEG Measures, below). For each participant and condition, mean PSD values were computed over two EEG frequency bands: theta/alpha (4–13 Hz) and beta (14–25 Hz). Mean PSD was computed over bilateral posterior scalp sites (PO7, PO8) demonstrating maximal PSD responses in each frequency band. These specific scalp sites and frequency bands were chosen based on the observed ranges of prominent differences in PSD of the resting EEG data (see Results, below).

### Computation of EEG complexity and integration

The complexity and integration measures utilized here are based in information theory, which views information as an ordered sequence of symbols. The information in a signal is then quantified in terms of its entropy *H*:

$$\begin{array}{l} H=\overset{N}{\underset{i=1}{\sum }}\;p_{i}\;\log_{2}\;p_{i} \end{array}$$

where *p*_*i*_ is the probability of occurrence of the *ith* possible value of a symbol. *H* indexes the uncertainty of a symbol sequence and thus how informative the sequence is. The integration *I(X)* and interaction complexity *C*_I_*(X)* of a set of signals X are then computed as Tononi et al. (1998a):

$$\begin{array}{l} I\left(X\right)=\overset{N}{\underset{i=1}{\sum }}H\left(X_{i}\right)-H\left(X\right)\\ C_{I}\left(X\right)=H\left(X\right)-\overset{N}{\underset{i=1}{\sum }}H\left(X_{i}|X-X_{i}\right) \end{array}$$

Here, *X*_*i*_ is the state of an individual EEG channel, *H(X*_*i*_*)* is the entropy of the channel, *H(X)* is the joint entropy of the coincident patterns *X* of binary states across all *N* EEG channels, and *H(X*_*i*_*|X–X*_*i*_*)* is the conditional entropy of a single EEG channel X_*i*_, given the state of the remaining multi-channels *X–X*_*i*_. If all EEG channels are statistically independent, then *I(X)* = 0, otherwise it is maximal when EEG activity is fully dependent across channels. In contrast, *C*_*I*_*(X)* is minimal for fully independent or dependent channels and is maximal when the channels are neither completely independent nor completely dependent; see Figure 1.

We computed the single-channel, joint, and conditional entropies of the EEG signals via explicit analytic expressions for the entropies based on the assumption that the EEG amplitudes realize continuous univariate and multivariate Gaussian processes with variances $\sigma_{\text{ii}}^{2}$ and covariance matrix *K* (Norwich, 1993; Tononi et al., 1994; van Putten and Stam, 2001; Ince et al., 2017):

$$\begin{array}{l} H\left(X_{i}\right)=\frac{1}{2\ln\;\left(2\right)}\cdot \ln\;\left(2\pi e\sigma_{ii}^{2}\right)\\ H\left(X\right)=\frac{1}{2\ln\;\left(2\right)}\cdot \ln\;\left\{{\left(2\pi e\right)}^{N}|K|\right\}\\ H\left(X_{i}|X-X_{i}\right)=H\left(X\right)-H\left(X-X_{i}\right) \end{array}$$

Continuous Gaussian univariate and multivariate entropies are *differential entropies* that are not independent of data units or scale; however, entropy differences—such as those that define *C*_I_*(X)*, *I(X)*, and *H(X_i_| X–X_i_)*—are data unit-/scale-independent (Norwich, 1993). All entropy functions were computed using the Information Breakdown Toolbox for MATLAB (Magri et al., 2009) with a correction for any information bias that may arise due to the estimation of the covariance matrices from limited data (Pola et al., 2003; Misra et al., 2005; Magri et al., 2009; Ince et al., 2017). The two mastoid sites could not be included in the entropy computations for the linked-mastoids reference due to the fact that, in this case, their linear dependence rendered the covariance matrix determinant |K| to be zero (Lay, 2005); hence, mastoid sites were also not included in the entropy calculations for the other EEG references in order to facilitate between-reference comparisons. All entropies were computed in terms of binary units (bits) of information.

Before computing the entropy functions, we first bandpass-filtered the resting EEG into two separate frequency ranges, the theta/alpha range (4–13 Hz) and the beta range (14–30 Hz), using a 424 point zero-phase shift FIR filter with 2 Hz transition bands. These specific frequency bands were chosen based on the observed ranges and scalp patterns of prominent differences in the spectral power of the resting EEG data (see Results, below). We performed this filtering step based on evidence that information-theoretic computations are more accurate when performed on data with a narrower frequency range (van Cappellen van Walsum et al., 2003).

Moreover, we took steps to ensure the filtered EEG signal distributions were approximately Gaussian, as assumed by the analytic expressions for H(X_*i*_), H(X), and H(X_*i*_|X–X_*i*_) above (see Discussion section for further elaboration of this requirement). First, we assessed the univariate and multivariate normality of the channels via Jarque-Bera tests (Jarque and Bera, 1987) and Royston's Test of Multivariate Normality (Royston, 1983), respectively, for each trial, condition, reference scheme, and participant. (Note that Royston's Test was computed via a publically available MATLAB script; Trujillo-Ortiz et al., 2007.) These tests indicated that, for the theta/alpha-range filtered data, the statistical distributions of approximately 49% of electrodes on average (collapsed across conditions and EEG reference schemes) violated the univariate normality assumption on any given trial, whereas the multivariate normality assumption was violated on 100% of trials on average. In the beta-range filtered data, the statistical distribution of 1% of electrodes on average (collapsed across conditions and EEG reference schemes) violated the univariate normality assumption on any given trial, whereas the multivariate normality assumption was violated on 99% of trials on average.

It is possible that our use of short (1 s), low sample number (256 samples) trials may have contributed, in part, to the rejection of the Gaussian-hypothesis for these trials. However, as will be argued in the Discussion section, we believe the pattern of results we observed in our data rule this out as a main origin of this rejection. In addition, the shorter EEG epochs utilized here better meet the assumptions of statistical stationarity, which is an important factor in the accurate assessment of the distribution of a time series data segment and EEG complexity (Branston et al., 2005); data epochs longer than 2 s yield poor assessments of goodness-of-fit (Elul, 1969). Use of short EEG trials also reduced the possibility that violations of stationarity across long data samples may distort complexity and integration calculations. (We note that we used 1-s trial lengths in order to minimize the number of trials that needed to be rejected due to muscle, signal, and ocular artifacts in the EEG. The latter were especially problematic, and we did not apply an ocular correction algorithm in order to avoid the possible effects of that algorithm on measurement of EEG complexity and integration.)

Hence, prior to calculation of C_I_(X) and I(X), we applied a method to transform non-normal distributions to Gaussian that have been successfully used before with EEG data (van Albada and Robinson, 2007). We transformed EEG data in this manner on a trial-by-trial basis for each separate frequency band, EEG reference, condition, and participant. After this transformation, we statistically assessed the univariate and multivariate normality of the channels again for each trial, condition, reference scheme, and participant. All tests were non-significant (*p* > 0.05, non-corrected) indicating that this Gaussian transformation process was successful. Inspection of the EEG waveforms and data histograms both before and after transformation showed that the transformation merely reduced the spread of outliers while simultaneously distributing the data more symmetrically around the mean. Importantly, we statistically-checked that this procedure did not did not distort the distributions of key EEG features of the eyes open and closed resting states (see Supplementary Material).

### Statistical analysis of EEG measures

We performed two kinds of statistical analysis of the EEG data, a parametric approach that assessed potential differences between EEG reference and resting state conditions and a non-parametric surrogate data testing approach that assessed the degree to which EEG complexity and integration may arise from random or spurious coincident activity among the EEG signals.

#### Parametric statistical approach

All parametric statistical analyses reported in this paper were performed using the SPSS software package (IBM Corporation, Armonk, NY, USA). Resting state EEG PSD, complexity, and integration were analyzed via repeated-measures ANOVA with within-participants factors of EEG Reference (LMR, AVG, INF, LAP) and Resting State Condition (Eyes Closed, Eyes Open). These analyses were performed separately for each frequency band. Given that the EEG Reference factor involved more than two levels, the *p*-values of all omnibus tests involving this factor were adjusted using the Greenhouse–Geisser correction for nonsphericity (Geisser and Greenhouse, 1958). For ease of interpretation, reports of all significant behavior *F* tests subject to Greenhouse-Geisser correction include uncorrected degrees of freedom, corrected *p*-values, and the Greenhouse-Geisser epsilon value ε. All *post-hoc* comparisons were corrected according to the Holm-Bonferroni procedure (Holm, 1979) and all corrected *p*-values are indicated as such in the text.

We also conducted regression analyses relating PSD to C_I_(X) and I(X) in order to quantify the relationship between these EEG metrics across individual participants. These regressions were conducted via generalized estimating equations (GEEs; Gardiner et al., 2009; Ma et al., 2012). GEEs are a generalized regression procedure that can account for the correlation structure across repeated measure levels while robustly estimating unbiased parameter standard errors. In the present study, the GEE analysis assumed a normal distribution with identity link, a robust covariance estimate, a maximum likelihood-estimate scale parameter, and an exchangeable correlation matrix. We conducted two regressions for each frequency band. The first regression was performed after collapsing across resting state conditions in order to assess the relationship between PSD and C_I_(X) and I(X) across EEG references. The second regression was performed after collapsing across EEG reference conditions in order to assess the relationship between the EEG metrics across resting state condition (eyes open, eyes closed). The reports of GEE analyses in this paper include standardized regression coefficients and tests of model effects (Wald χ*2* statistic values, associated degrees of freedom, *p*-values).

#### Surrogate data testing

It is well-known that random or spuriously-coincident EEG activity may produce apparent statistical dependencies among otherwise independent EEG sources, with this effect exacerbated by volume conduction (Nunez et al., 1997; Lachaux et al., 1999, 2000). In order to estimate the level of spurious complexity and integration in our EEG data that may arise from volume conduction, we created surrogate EEG data with similar statistical characteristics and spectral power distributions as the observed data, but that arise from a superposition of statistically independent sources with randomly-shifted EEG signal phases. This was achieved using a modification of the method of Shahbazi et al. (2010). In this method, each observed data set is first decomposed via Independent Components Analysis (ICA; Lee et al., 1999) to create signals that are independent as possible (ICA creates decompositions of nearly, but not perfectly, independent signals). In a second step, any remaining statistical dependencies are destroyed by randomly shifting the nth ICA component activation time course by a time (*n*–1)^*^ T, where T is larger than any autocorrelation time (practically, T must be a least one trial length). In a third step, the shifted ICA activations are then transformed back into EEG sensor space via the ICA inverse mixing matrix. In the present study, we implemented the second step for each surrogate trial by randomly sampling from the remaining trials for each ICA activation time course. This sampling was performed without replacement to ensure that none of the ICA activation signals came from the same trial; this yielded effective values for T ranging from one to several hundred trial lengths, depending on the number of trials for a given data set.

We computed distributions of complexity and integration values from 100 surrogate data sets created for each subject, condition, and EEG frequency band. We then computed the mean and two-tailed 95% confidence intervals (CIs) of these distributions. Observed mean complexity or integration values that were found to lie outside of these confidence intervals were interpreted as being significantly different from any complexity/integration that may arise purely from random or spuriously coincident volume-conducted EEG activity. Surrogate data testing was performed via in-house MATLAB scripts. ICA decompositions were implemented using the extended infomax runica algorithm (with data whitening and default stopping weight change = 1e-07) implemented within the EEGLAB toolbox for MATLAB (Delorme and Makeig, 2004).

### Dipole modeling

**Figure 3:**
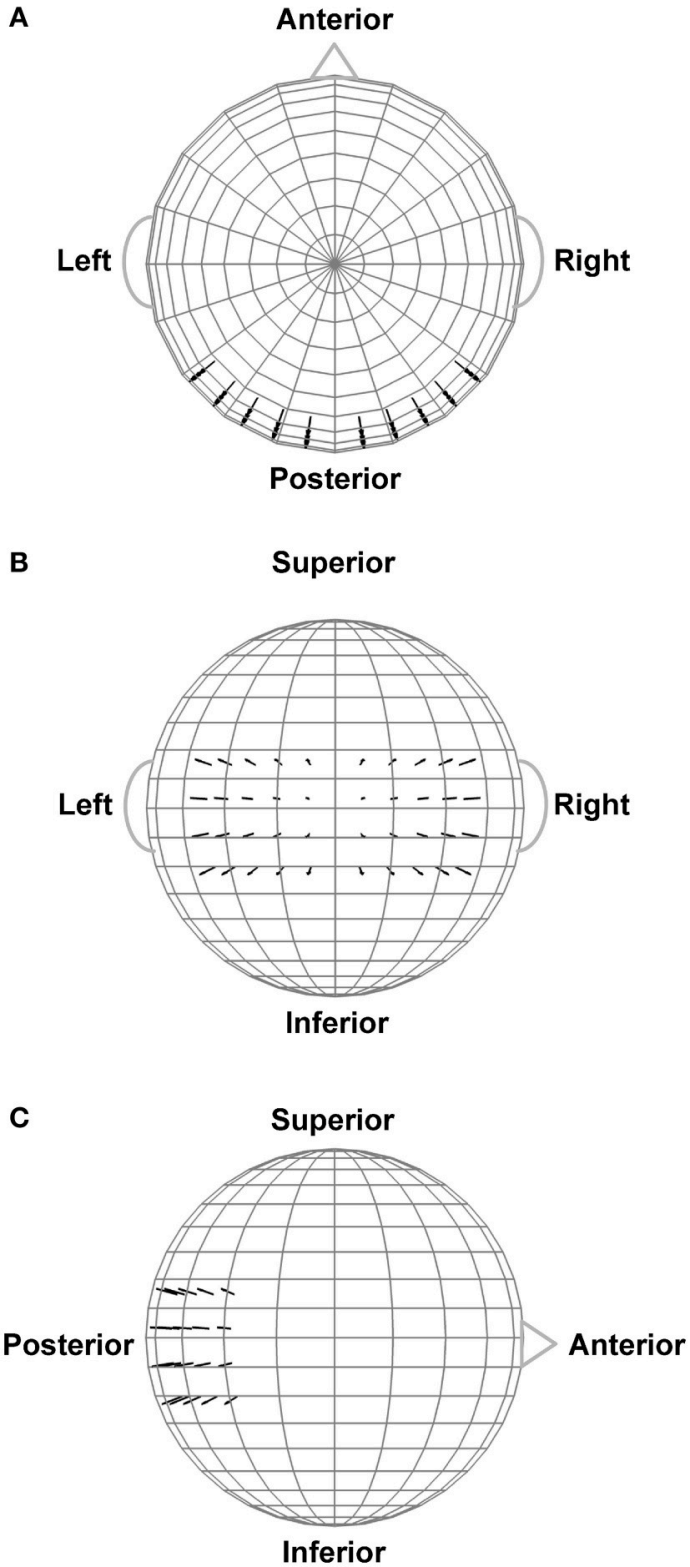
Extrastriate dipole sources (black arrows) positioned within a 4-shell spherical head forward volume-conduction model (only the outer shell surface is represented in the figure). The remainder of the spherical surface was filled with 148 equally-spaced background activity dipoles (not shown). All dipoles were placed at a superficial (2 mm subdural) cortical locations. **(A)** Top head view. **(B)** Rear head view. **(C)** Side head view.

In order to assist interpretation of the observed EEG complexity and integration, we simulated oscillatory EEG data at the scalp from oscillating, fixed-location intracranial dipole sources with pre-determined complexity and integration. The dipole sources were positioned within a concentric 4-shell spherical head forward volume-conduction model (Cuffin and Cohen, 1979; Mosher et al., 1993; Tenke and Kayser, 2015) implemented via in-house MATLAB scripts. The model's outer shell had an 85 mm radius, with a scalp thickness = 3 mm (σ_scalp_ = 0.33 Ω/m), bone thickness = 4 mm (σ_bone_ = 0.0042 Ω/m), CSF layer = 2 mm (σ_CSF_ = 1 Ω/m), and a brain surface at a 76 mm radius (σ_brain_ = 0.33 Ω/m); shell thickness and conductivity values were taken from Cuffin and Cohen (1979) and Mosher et al. (1993). Simulated scalp electrode locations were the same as for the EEG recordings (see Figure 2). We created two clusters of 20 radially-oriented dipole generators (40 dipoles total) at posterior, roughly extrastriate cortical locations (Figure 3), one cluster in each hemisphere with the anterior-posterior location of both clusters centered on the equatorial (FPZ-T7-T8-OZ) plane. These *extrastriate dipoles* were used to simulate posterior cortical processes known to be active during EEG resting tasks (Feige et al., 2005). The remainder of the spherical surface was filled with 148 equally spaced dipoles to simulate background cortical processes (termed here *background dipoles*). All dipoles were placed at superficial cortical locations (2 mm subdural, following Tenke and Kayser, 2015).

The magnitude of each dipole source moment varied sinusoidally over time characterized by an amplitude, frequency, and phase chosen according to a particular simulated resting state EEG condition (see below). We set the frequency ranges of these oscillations to be those analyzed for the empirical data (theta-alpha range: 4–13 Hz; beta range: 14–30 Hz); phases were uniformly distributed from 0 to 2π. The frequency and phase of each sinusoid waveform were randomly drawn with replacement (1 Hz frequency resolution) from these ranges for each simulated trial, subject to specific interdipole dependency relationships (see below). Background dipoles had a dipole source moment of 1 μA-cm for the theta-alpha range simulations and 0.50 μA-cm for the beta range simulations. For the extrastriate dipoles, we simulated potential amplitude differences between eyes closed and open resting state conditions by performing one set of simulations where the maximum magnitude of each dipole source moment was high (theta-alpha range: 1.25 μA-cm; beta-range: 0.625 μA-cm) and a second set of simulations where the maximum magnitude of each source was low (theta-alpha range: 1.00 μA-cm; beta-range: 0.50 μA-cm). Moreover, fluctuations in ongoing EEG amplitudes were modeled by multiplying simulated dipole moment time courses by a Gaussian window (σ_*t*_ = 250 ms) whose temporal center randomly varied along the time dimension of each epoch. The Gaussian window multiplication reproduced amplitude modulations in the observed data. Window locations were different on each trial, and were different for independent sources, but the same for dependent sources (see interdipole dependencies, below). Window locations and spread were the same for all interdipole dependency and dipole amplitude conditions. These amplitude modulations introduced an extra degree of randomness into the simulations that affected the overall magnitude of complexity and integration, but did not affect the general pattern of results observed here. One hundred trials were created in each simulation; each trial was 1 s long.

Scalp potential topographies and time courses were simulated for each dipole generator separately. In order to assess the complexity and integration of the dipole moment time series, the dipole moment waveforms underwent the same Gaussian transformation procedure as the empirical EEG data; the transformed dipole moments were also used to create the forward volume-conductions to the scalp. The linearity of volume conduction allowed the final simulated EEG scalp record to be constructed from the sum of the individual dipole topographies at each time point (Tenke and Kayser, 2015). We then re-referenced the simulated scalp EEG signals according to the four EEG references investigated in this study. The scalp level time series for each reference also underwent Gaussian transformation before computation of EEG complexity, integration, and spectral power.

We created several different models that differed in terms of the temporal dependencies—and thus the complexity and integration—among the extrastriate dipole moment waveforms. These models were created by manipulating the shared frequencies and phases of each dipole waveform on any given trial as follows: (1) all dipoles shared the same frequency, phase, and amplitude fluctuation patterns on any given trial (*full dependency model*); (2) shared frequency/phase/amplitude fluctuations for 75% of the dipoles, independent frequencies/phases/amplitude fluctuations for the remaining dipoles (*interdependent model—level 1*); (3) shared frequency/phase/amplitude fluctuations for 50% of the dipoles, independent frequencies/phases/amplitude fluctuations for the remaining dipoles (*interdependent model—level 2*); (4) shared frequency/phase/amplitude fluctuations for 25% of the dipoles, independent frequencies/phases/amplitude fluctuations for the remaining dipoles (*interdependent model—level 3*); (5) all dipole waveforms independent in frequency/phase and amplitude fluctuations, but with the same phase for each time point (*independent model—level 1*); (6) all dipole waveforms initially created with independent frequencies/phases/amplitude fluctuations, but then phases randomized further via a FFT-based procedure (Theiler et al., 1992) that reduces the autocorrelation of a signal (*independent model—level 2*); and (7) dipole waveforms composed of multivariate Gaussian noise with an identity covariance matrix (*full independency model*). Although the additional phase randomization step used for the level-2 independent model may seem redundant, we found that it further increased the independence of the dipole moment waveforms (see Results, below), most likely due to a reduction of signal autocorrelation. In contrast to the extrastriate dipoles, all background dipole waveforms were given level-2 independent model temporal dependencies in order to simulate random background noise within a frequency range of interest.

## Results

All empirical data and MATLAB data analysis and dipole simulation scripts are available online at the Texas State University Data Repository (Trujillo et al., 2017; https://dataverse.tdl.org/dataverse/rsed2017).

### Power spectral density (PSD)

**Figure 4:**
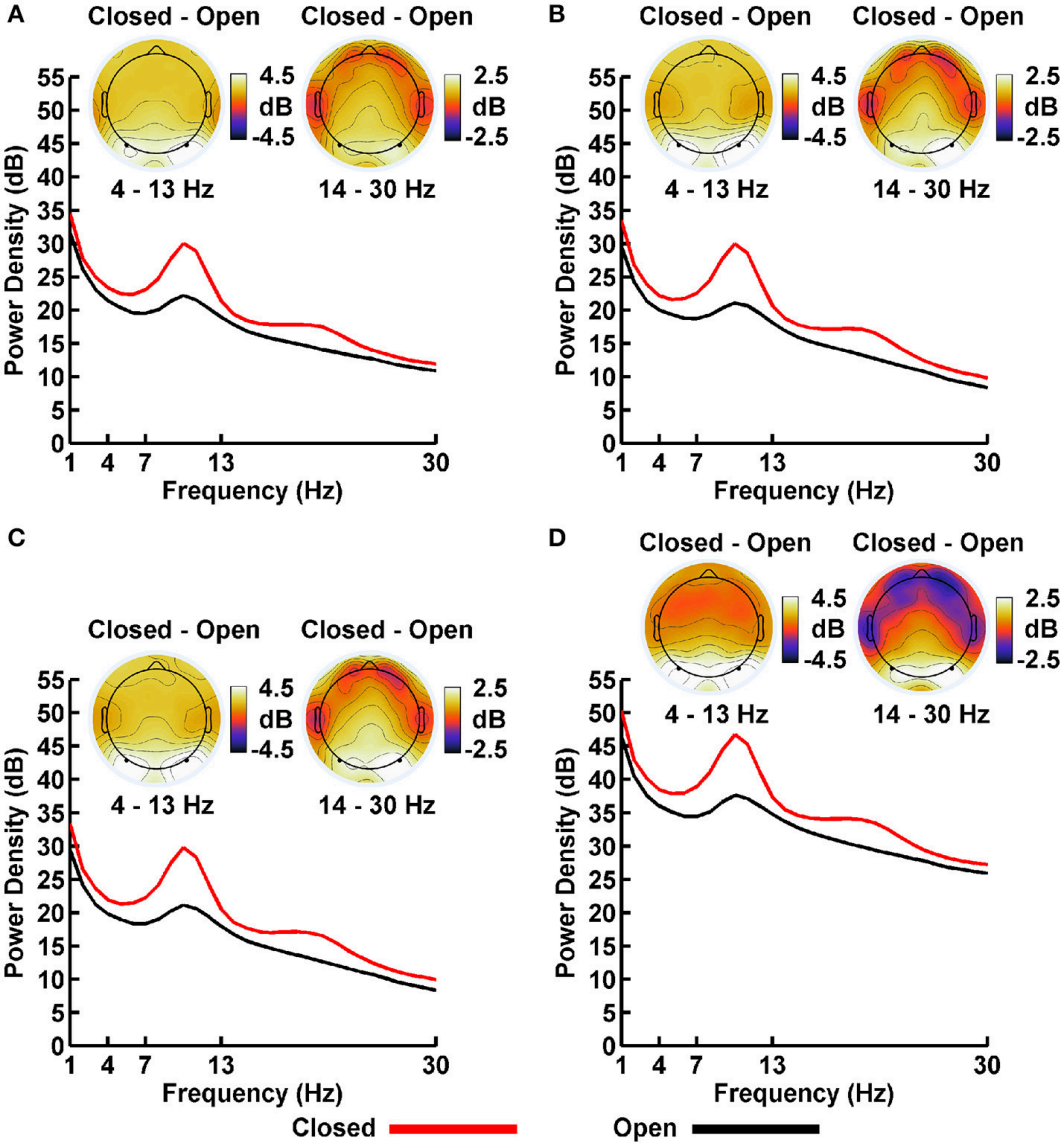
Resting EEG spectral power density (PSD, in decibels) for eyes closed (red line) and eyes open (black line) conditions for **(A)** LM, **(B)** AVG, **(C)** INF, and **(D)** LAP reference schemes. Power spectra are collapsed across a priori electrodes of interest indicated by black/white circles on the head maps (see Methods). Head maps display power difference topographies averaged over the stated frequency intervals; light/dark colors indicate ± values.

Plots of non-Gaussian-transformed resting state EEG PSD are shown in Figure 4; mean power values are listed in Table 1 (mean PSD values for Gaussian-transformed data are listed in Table S1). Preliminary analysis (not shown) indicated that the posterior topographical distributions of resting-state theta- and alpha-PSD differences were nearly identical, thus justifying the collapse across these two frequency bands for statistical analysis and data presentation. In contrast, beta-range PSD had a slightly more anterior topographical distribution relative to the theta-alpha range.

**Table 1:** Mean EEG power spectral density by EEG frequency band, resting state condition, and EEG reference.

**EEG band**	**EEG reference**	**Eyes closed**	**Eyes open**
Theta/Alpha	*LM*	24.95 (0.60)	20.50 (0.53)
	*AVG*	24.35 (0.63)	19.55 (0.54)
	*INF*	24.16 (0.64)	19.34 (0.55)
	*LAP*	40.91 (0.68)	35.74 (0.62)
Beta	*LM*	16.95 (0.53)	14.57 (0.44)
	*AVG*	16.00 (0.55)	13.26 (0.42)
	*INF*	15.91 (0.54)	13.13 (0.41)
	*LAP*	32.95 (0.61)	30.03 (0.50)

*All values are in dB; SE in parentheses*.

For both frequency ranges, EEG PSD was largest for the LAP transformation, followed by the LM reference, AVG reference, and INF reference [Theta/Alpha: *F*_(3, 63)_ = 7,046.32, *p*__GG−corrected_ < 0.001, ε = 0.57, η^2^_*P*_ = 0.99; Beta: *F*_(3, 63)_ = 4,551.63, *p*__GG−corrected_ < 0.001, ε = 0.63, η^2^_*P*_ = 0.99; all *post*-*hoc p*s__HB−corrected_ < 0.048]. Furthermore, EEG PSD was larger for the eyes closed vs. eyes open resting state condition across both frequency ranges [Theta/Alpha: *F*_(1, 21)_ = 204.18, *p*__GG−corrected_ < 0.001, η^2^_*P*_ = 0.91; Beta: *F*_(1, 21)_ = 85.29, *p*__GG−corrected_ < 0.001, η^2^_*P*_ = 0.80]. However, EEG Reference × Resting State Condition interactions were significant for both frequency bands [Theta/Alpha: *F*_(3, 63)_ = 28.77, *p*__GG−corrected_ < 0.001, ε = 0.68, η^2^_*P*_ = 0.58; Beta: *F*_(3, 63)_ = 12.51, *p*__GG−corrected_ < 0.001, ε = 0.70, η^2^_*P*_ = 0.37; all *post-hoc p*s__HB−corrected_ < 0.004]. Decomposition of the interaction for the theta/alpha frequency band indicated that the resting state eyes closed vs. eyes open PSD differences were largest for the LM reference (4.45 ± 0.30 dB), followed by the LAP transformation (5.17 ± 0.38 dB), and then the AVG (4.80 ± 0.33 dB) and INF (4.82 ± 0.35 dB) references, *p*s__corrected_ < 0.006, although the resting state condition PSD relative to the AVG and INF references did not significantly differ from each other in this frequency band, *p*__corrected_ > 0.525. Decomposition of the interaction for the beta frequency band indicated that the resting state eyes closed vs. eyes open PSD difference was larger for the LM reference (2.38 ± 0.27 dB) vs. the other reference schemes (LAP: 2.92 ± 0.33 dB; AVG: 2.74 ± 0.30 dB; INF: 2.78 ± 0.29 dB), *p*s__corrected_ < 0.006, whereas beta-range PSD differences were not significantly different among the LAP, AVG, and INF schemes, *p*s__corrected_ > 0.215; see Table 1.

### EEG complexity and integration

Mean observed resting EEG interaction complexity C_I_(X) and integration I(X) values are given in the left columns of Tables 2, 3; ANOVA results are given in Table 4.

**Table 2:** Mean resting EEG complexity by EEG frequency band, resting state condition, and EEG reference.

**EEG band**	**EEG reference**	**Observed data**		**Surrogate data**	
		**Eyes closed**	**Eyes open**	**Eyes closed**	**Eyes open**
Theta/Alpha	*LM*	67.15 (0.52)	70.90 (0.43)	69.23 (67.41,71.05)	72.33 (70.61,74.05)
	*AVG*	69.84 (0.65)	74.19 (0.59)	73.38 (71.18,75.58)	77.88 (75.64,80.12)
	*INF*	69.62 (0.63)	74.10 (0.58)	73.34 (71.14,75.54)	77.53 (75.38,79.68)
	*LAP*	78.60 (0.37)	80.76 (0.31)	100.58 (99.53,101.63)	103.17 (102.28,104.06)
Beta	*LM*	78.12 (0.78)	81.59 (0.69)	77.64 (75.32,79.95)	80.04 (77.49,82.59)
	*AVG*	84.77 (0.94)	89.30 (0.92)	85.68 (83.05,88.30)	89.57 (86.97,92.17)
	*INF*	84.27 (0.89)	88.71 (0.84)	84.85 (82.39,87.31)	88.21 (85.87,90.54)
	*LAP*	92.57 (0.47)	93.29 (0.57)	109.56 (108.36,110.75)	109.82 (108.25,111.40)

*All values are in bits; SE in parentheses for observed data, 95% CIs in parentheses for surrogate data*.

**Table 3:** Mean resting EEG integration by EEG frequency band, resting state condition, and EEG reference.

**EEG band**	**EEG reference**	**Observed data**		**Surrogate data**	
		**Eyes closed**	**Eyes open**	**Eyes closed**	**Eyes open**
Theta/Alpha	*LM*	197.75 (2.15)	184.77 (1.42)	189.37 (183.24,195.51)	175.51 (171.04,179.99)
	*AVG*	179.16 (1.79)	167.71 (1.41)	169.84 (164.48,175.19)	157.95 (153.02,162.88)
	*INF*	180.95 (1.75)	168.94 (1.38)	171.77 (166.57,176.97)	159.18 (154.40,163.95)
	*LAP*	156.53 (0.66)	152.70 (0.55)	138.33 (135.61,141.04)	133.33 (130.66,136.01)
Beta	*LM*	173.50 (2.01)	165.66 (1.84)	167.32 (162.28,172.36)	158.58 (153.90,163.26)
	*AVG*	152.84 (1.57)	145.10 (1.40)	146.08 (141.47,150.68)	137.57 (133.51,141.64)
	*INF*	154.31 (1.52)	146.23 (1.30)	147.17 (142.78,151.55)	138.17 (134.52,141.82)
	*LAP*	142.27 (0.74)	141.39 (1.08)	129.85 (127.35,132.34)	128.60 (125.24,131.96)

*All values are in bits; SE in parentheses for observed data, 95% CIs in parentheses for surrogate data*.

**Table 4:** Analysis of variance (ANOVA) results for EEG complexity and integration for each frequency band (see Material and Methods—Computation of EEG Complexity and Integration).

**EEG band**	**EEG measure**	**Effect**	**F**	**p**	**ε**	**η^2^_*P*_**
Theta/Alpha	*C_*I*_*(X)	*REF*	662.80	^†^0.001	0.55	0.97
		*RS*	133.19	0.001	–	0.86
		*REF* × *RS*	96.12	^†^0.001	0.53	0.82
	
	*I*(X)	*REF*	468.32	^†^0.001	0.65	0.96
		*RS*	100.86	0.001	–	0.83
		*REF × RS*	64.25	^†^0.001	0.51	0.75
Beta	*C_*I*_*(X)	*REF*	39.59	^†^0.001	0.68	0.87
		*RS*	96.85	0.105	–	0.82
		*REF × RS*	92.64	^†^0.041	0.63	0.82
	
	*I*(X)	*REF*	114.96	^†^0.001	0.52	0.85
		*RS*	67.18	0.001	–	0.76
		*REF × RS*	91.01	^†^0.001	0.58	0.81

*ANOVA factor labels: REF, EEG Reference; RS, EEG Resting State. REF factor effects dfs = 3, 63; RS main effect df = 1, 21*.

† *The symbol indicates p-values subject to Greenhouse-Geisser correction (see Materials and Methods—Statistical Analysis of EEG/ERP Measures section)*.

In the theta/alpha frequency range, C_I_(X) was significantly largest for the LAP transformation (79.68 ± 0.32 bits), smallest for the LM reference (69.03 ± 0.45 bits), with the AVG reference (72.06 ± 0.59 bits) and INF reference (71.86 ± 0.57 bits) taking intermediate values (see Figure 3A); all between-reference C_I_(X) differences were significant, *post-hoc p*s__corrected_ < 0.006. A main effect of resting state condition indicated that C_I_(X) was larger for eyes open (75.01 ± 0.46 bits) than the eyes closed condition (71.30 ± 0.53 bits) collapsed across EEG references in this frequency range (Table 2 and Figure 3A). Decomposition of the significant EEG Reference × Resting State Condition interaction (Table 2) indicated that the theta/alpha-range eyes open vs. eyes closed C_I_(X) differences were larger for the INF (4.48 ± 0.38 bits) and AVG (4.45 ± 0.37 bits) references vs. the LAP transformation (2.16 ± 0.24 bits) and LM reference (3.75 ± 0.33 bits), *p*s__corrected_ < 0.006. Eyes closed vs. eyes open complexity was also significantly different between the LAP transformation and LM reference, *p*__corrected_ < 0.006, but not between the INF and AVG references, *p*__corrected_ = 0.432; see Table 2.

Theta/alpha range I(X) followed an opposite pattern than complexity, being significantly smallest for the LAP transformation (154.61 ± 0.57 bits), largest for the LM reference (191.26 ± 1.66 bits), with the INF reference (174.95 ± 1.47 bits) and AVG reference (173.43 ± 1.52 bits) taking intermediate values (see Figure 3A); all between-reference C_I_(X) differences were significant, *post-hoc* ps__corrected_ < 0.006. A main effect of resting state condition indicated that I(X) was larger for eyes closed (178.60 ± 1.52 bits) than the eyes open (168.53 ± 1.11 bits) condition across EEG references in this frequency range (Table 2 and Figure 3A). However, decomposition of the significant EEG Reference x Resting State Condition interaction (Table 2) indicated that the magnitude of the resting state eyes closed vs. eyes open integration differences were smallest for the LAP transformation (3.83 ± 0.4 bits) vs. the other references (LM: 12.98 ± 1.50 bits; AVG: 11.46 ± 1.07 bits; INF: 12.01 ± 1.12 bits), ps__corrected_ < 0.006, whereas these integration differences were not significantly different among the LM, AVG, and INF references, *p*s__corrected_ > 0.09; see Table 3.

In the beta frequency range, C_I_(X) was significantly largest for the LAP transformation (92.93 ± 0.50 bits), followed by the AVG reference (87.03 ± 0.91 bits), INF reference (86.49 ± 0.84 bits), and LM reference (79.85 ± 0.72 bits), *post-hoc p*s__corrected_ < 0.006; see Figure 3B. Additionally, C_I_(X) was larger in the eyes open (88.22 ± 0.63 bits) vs. eyes closed (84.93 ± 0.71 bits) resting state conditions for C_I_(X) collapsed across EEG references (see Table 2). Decomposition of the significant EEG Reference x Resting State Condition interaction (Table 2) indicated that the beta resting state eyes open vs. eyes closed C_I_(X) differences were larger for the INF (4.44 ± 0.40 bits) and AVG (4.53 ± 0.42 bits) references vs. the LAP transformation (3.48 ± 0.35 bits) and LM reference (0.72 ± 0.30 bits), *p*s__corrected_ < 0.006. Eyes closed vs. eyes open complexity was also significantly different between the LAP transformation and LM reference, *p*__corrected_ < 0.006, but not between the INF and AVG references, *p*__corrected_ = 0.203; see Table 2.

Beta-range I(X) followed an opposite across-reference pattern than complexity, being significantly smallest for the LAP transformation (141.83 ± 0.87 bits), largest for the LM reference (169.58 ± 1.87 bits), with the INF reference (150.26 ± 1.34 bits) and AVG reference (148.97 ± 1.43 bits) taking intermediate values (see Figure 3B); all between-reference C_I_(X) differences were significant, *post-hoc p*s__corrected_ < 0.006. A main effect of resting state condition indicated that I(X) was larger for eyes closed (155.73 ± 1.22 bits) than the eyes open (149.59 ± 1.01 bits) condition across EEG references in this frequency range (Table 2 and Figure 3B). However, decomposition of the significant EEG Reference x Resting State Condition interaction (Table 2) indicated that the magnitude of the resting state eyes closed vs. eyes open integration differences were smaller for the LAP transformation (0.88 ± 0.61 bits) vs. the other references (LM: 7.84 ± 0.92 bits; AVG: 7.73 ± 0.83 bits; INF: 8.08 ± 0.86 bits), *p*s__corrected_ < 0.006, and smaller for the AVG vs. INF reference, *p*__corrected_ < 0.006. The integration differences between the LM and AVG/INF references were not significant different, *p*s__corrected_ > 0.589; see Table 3.

Finally, the GEE-based regression analysis examining the relationship between PSD and C_I_(X) and I(X) across EEG references (collapsed across resting state condition) showed that theta-alpha PSD was positively associated with C_I_(X), β = 0.94 ± 0.02, Wald χ^2^_(1, 22)_ = 1,481.87, *p* < 0.001, but negatively associated with I(X), β = −0.72 ± 0.03, Wald $\chi_{\left(1,\;22\right)}^{2}$ = 579.93, *p* < 0.001. Similarly, beta PSD was positively associated with C_I_(X), β = 0.55 ± 0.07, Wald $\chi_{\left(1,\;22\right)}^{2}$ = 64.03, *p* < 0.001, but negatively associated with I(X), β = −0.31 ± 0.07, Wald χ^2^_(1, 22)_ = 19.32, *p* < 0.001. Thus, the increase in EEG power across EEG reference schemes corresponded to an increase in EEG complexity and decrease in EEG integration. The regression analysis examining the relationship between PSD and C_I_(X) and I(X) across resting state conditions (collapsed across EEG reference) showed that theta-alpha PSD was negatively associated with C_I_(X), β = −0.95 ± 0.04, Wald $\chi_{\left(1,\;22\right)}^{2}$ = 477.46, *p* < 0.001, but positively associated with I(X), β = 0.96 ± 0.05, Wald $\chi_{\left(1,\;22\right)}^{2}$ = 390.93, *p* < 0.001. Similarly, beta PSD was negatively associated with C_I_(X), β = −0.86 ± 0.05, Wald $\chi_{\left(1,\;22\right)}^{2}$ = 346.63, *p* < 0.001, but positively associated with I(X), β = 0.94 ± 0.04, Wald $\chi_{\left(1,\;22\right)}^{2}$ = 516.04, *p* < 0.001. Thus, the increase in EEG power across resting state conditions corresponded to a decrease in EEG complexity and an increase in EEG integration.

### Surrogate data tests

Mean surrogate resting EEG interaction complexity C_I_(X) and integration I(X) values are given in the right columns of Tables 2, 3. It is clear from the tables that, in the theta-alpha frequency range, all observed mean complexity values lay outside the surrogate 95% two-tailed confidence intervals for all four EEG references in the eyes closed condition, and for the AVG, INF, and LAP references in the eyes open condition. In the beta range, only the LAP reference yielded complexity values outside the surrogate confidence intervals for either resting state condition. All EEG references yielded mean integration values outside the surrogate confidence intervals for both resting state conditions and frequency ranges. It is unlikely that the observed complexity and integration values outside the surrogate confidence intervals are attributable to spurious interactions.

**Figure 5:**
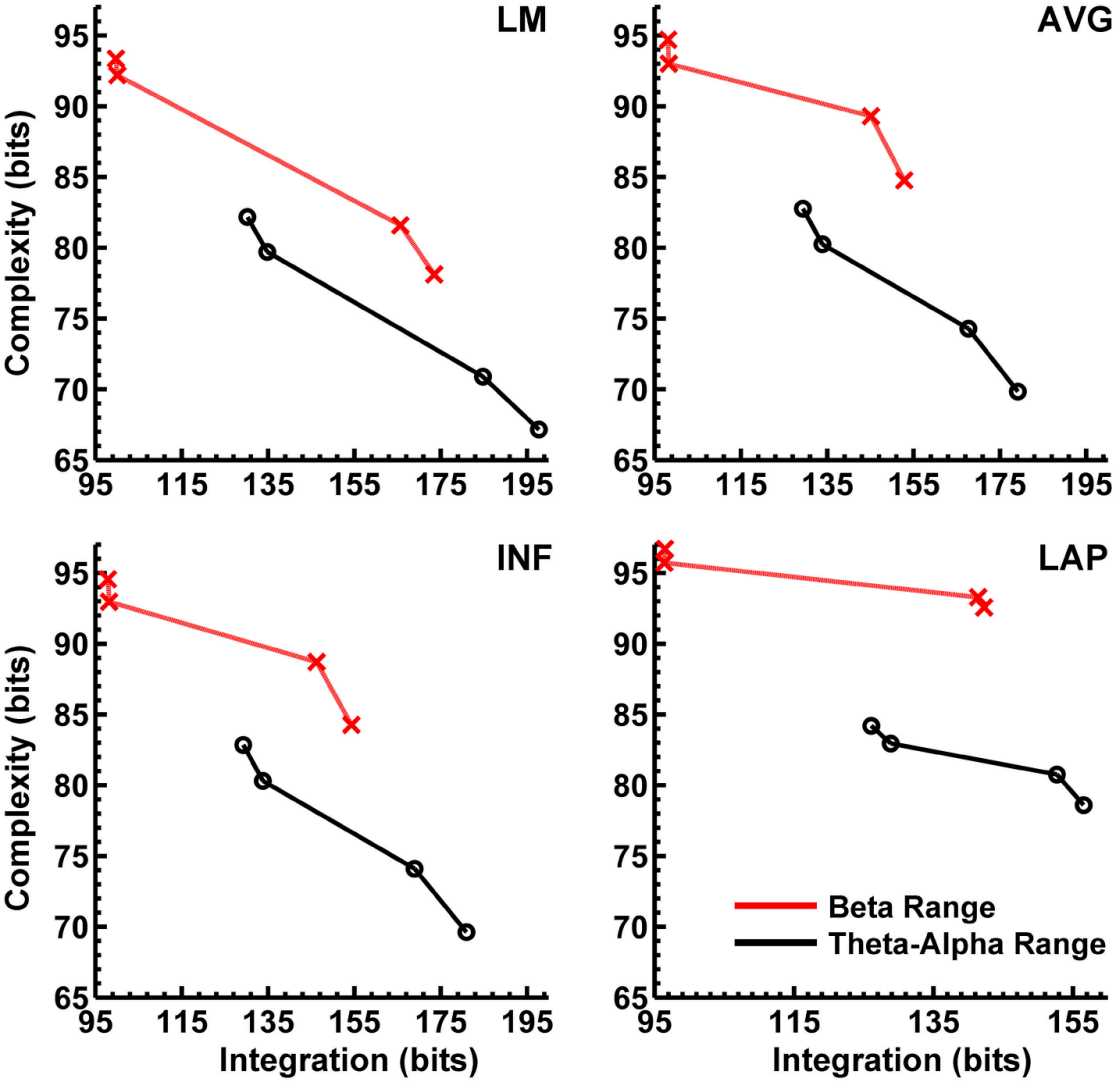
Empirical interaction complexity C_I_(X) as a function of integration I(X) for observed and surrogate scalp EEG. Data points are ordered in increasing dependency from left to right (eyes open surrogate, eyes closed surrogate, eyes open observed, eyes closed observed). Red lines, beta-range data; black lines, theta-alpha-range data. Data points reflect averages across 22 participants; standard errors of data points (not shown) are given in Tables 2, 3.

The observed mean integration values tended to be larger than the surrogate values, whereas the observed mean complexity values tended to be lower than the surrogate values. This pattern in integration and complexity is consistent with the theoretical prediction (Tononi et al., 1994; see Figure 1) that as system elements in the high integration regime become more independent (in this case, artificially via the surrogate data creation procedure), C_I_(X) should increase and I(X) should decrease. Indeed, this predicted relationship becomes apparent when the eyes closed and eyes open observed and surrogate EEG data are ordered in terms of monotonically increasing integration values (Figure 5).

### Dipole modeling

**Figure 6:**
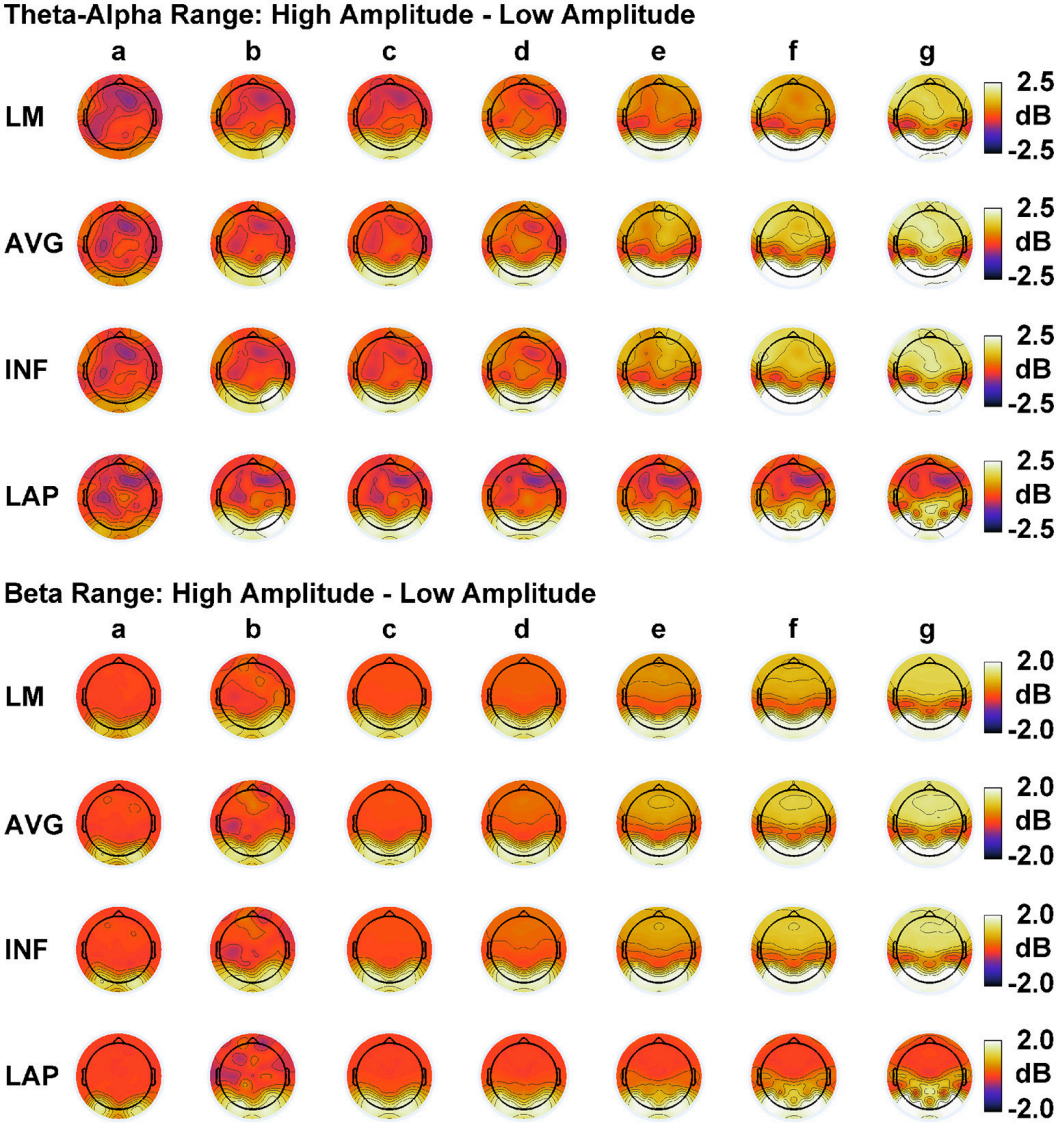
Simulated resting state EEG. Scalp topographies show high amplitude—low amplitude PSD differences over the theta-alpha frequency range **(top)** and beta frequency range **(bottom)**. PSD topographies are generated from seven different interdipole dependency models (in increasing dependency from left to right): **(a)** full independency model, **(b)** independent model—level 2, **(c)** independent model—level 1, **(d)** interdependent model—level 3, **(e)** interdependent model—level 2, **(f)** interdependent model—level 1, and **(g)** full dependency model. Head maps display PSD topographies averaged over the stated frequency ranges; light/dark colors indicate ± values. Scalp maps reflect averages across 22 separate simulations.

**Figure 7:**
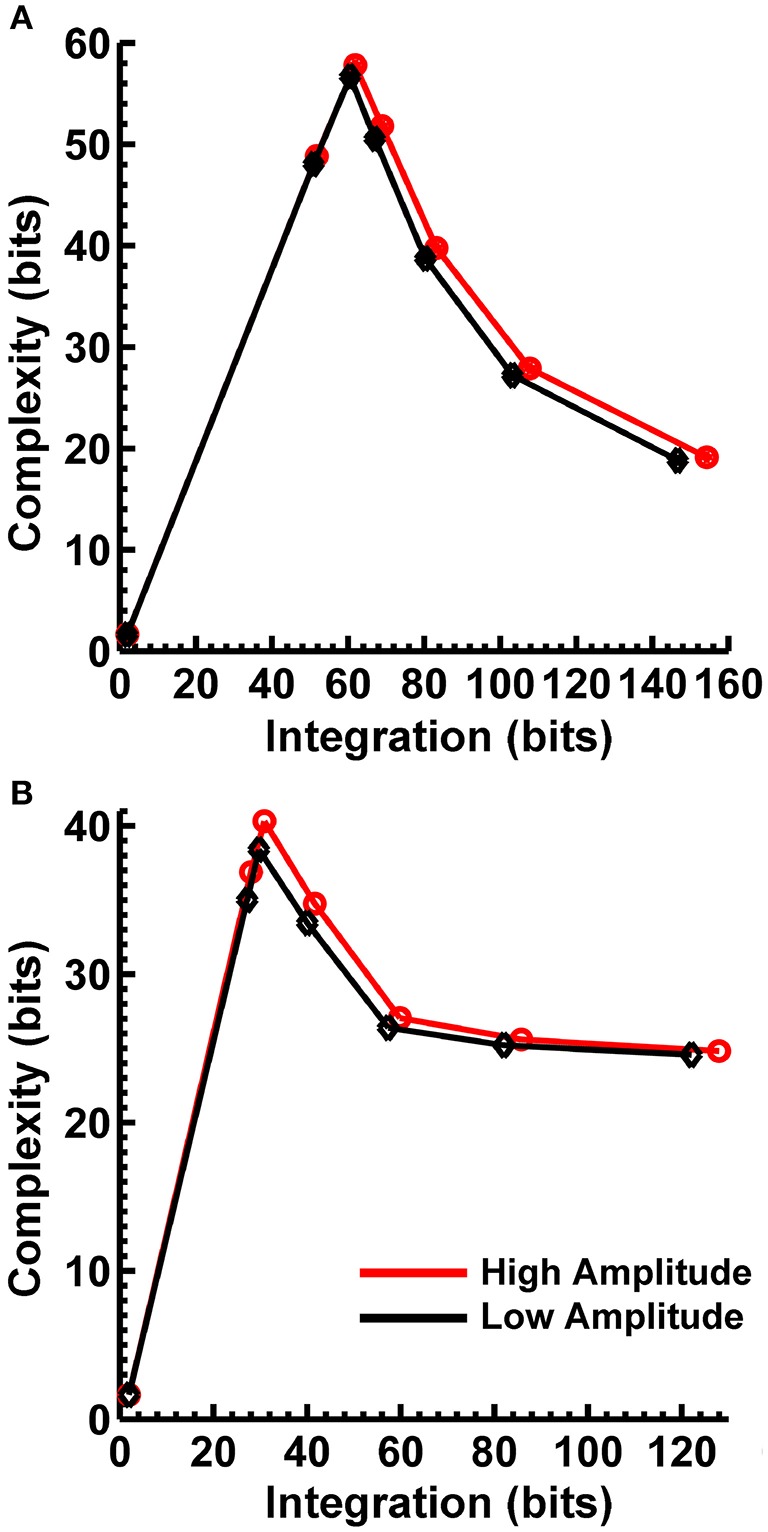
Interaction complexity C_I_(X) as a function of integration I(X) for simulated **(A)** theta-alpha range and **(B)** beta-range visual dipole moment sources (excluding background sources). Data points are generated from the seven different interdipole dependency models and are ordered from left to right in terms of increasing dependency/integration (full independency model, independent model—level 2, independent model—level 1, interdependent model—level 3, interdependent model—level 2, interdependent model—level 1, full dependency model). Red lines, high amplitude simulations; black lines, low amplitude simulations. Data points reflect averages across 22 separate simulations; standard errors of simulated C_I_(X) and I(X) are too small to be displayed but range from 0.01 to 0.21 bits.

In order to match the number of participants in the observed data and to ensure that our modeling results did not depend on one specific set of randomly determined parameters, we created 22 separate simulations for each specific model and then averaged across each set of simulations and trials within a simulation for a given model. Figure 6 displays mean simulated resting state EEG PSD differences between the high vs. low amplitude simulations for the theta-alpha and beta frequency ranges. The qualitative features of the simulated topography differences are in good agreement with the difference topographies of the observed data, although this agreement is reduced somewhat for the full interdipole dependency simulations. Figure 7 shows mean C_I_(X) as a function of mean I(X) for simulated extrastriate dipole moment sources only (not including the background sources). The simulated relationship between complexity and integration is in agreement with theoretical predictions (e.g., Figure 1). C_I_(X) and I(X) are low for the full independency model when the extrastriate dipoles are statistically independent. C_I_(X) and I(X) increase for level-2 independence, with C_I_(X) achieving a maximum for level-1 independence of the extrastriate dipoles at intermediate values of I(X). As integration continues to increase, C_I_(X) decreases through the three increasing levels of interdependency among the extrastriate dipoles, reaching a local minimum for the fully dependent extrastriate dipoles. In addition, Figure 7 also shows that C_I_(X) and I(X) tended to be greater for the high amplitude dipole moments vs. the low amplitude moments at higher levels of statistical dependence among the extrastriate dipoles.

**Figure 8:**
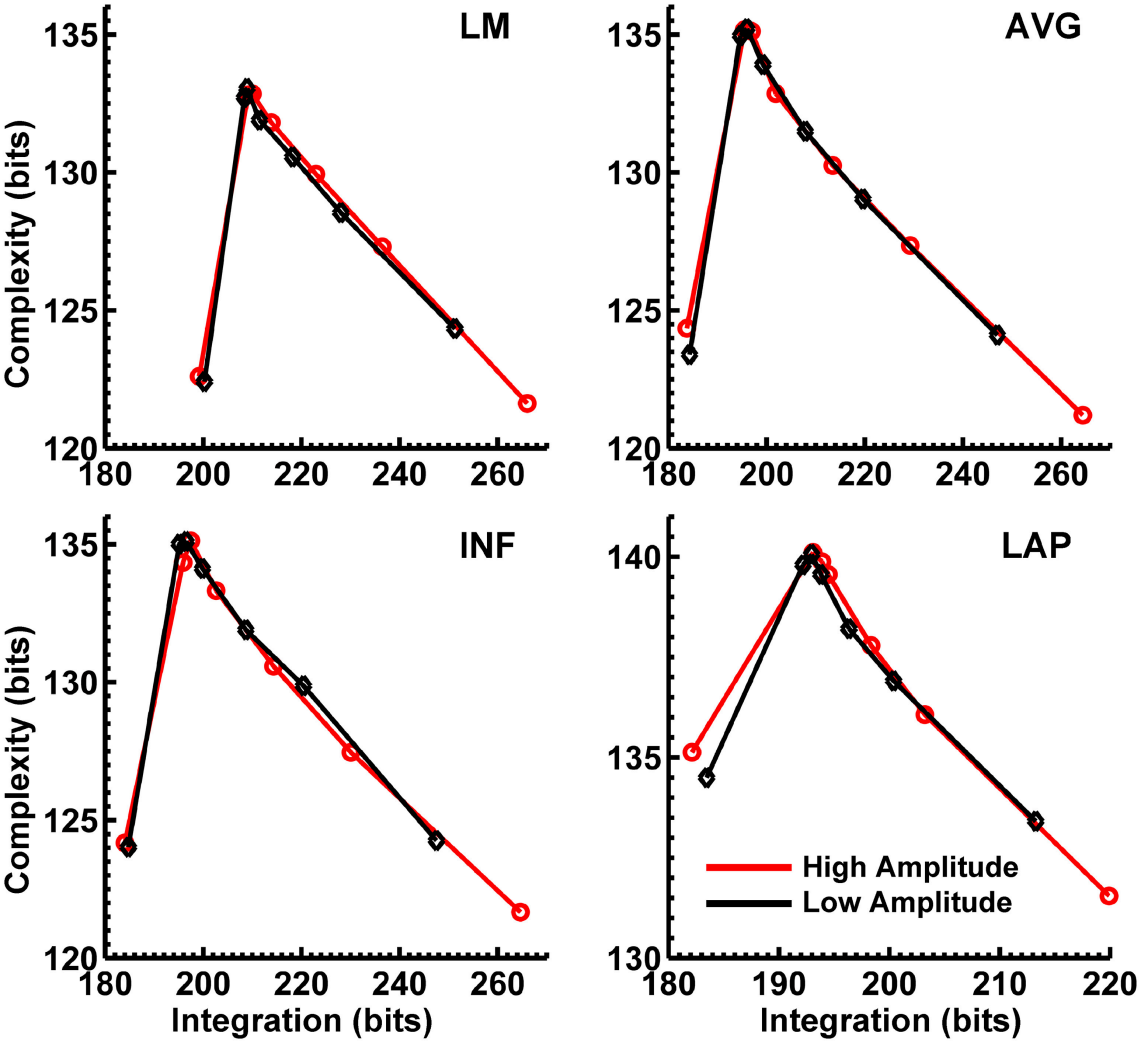
Interaction complexity C_I_(X) as a function of integration I(X) for theta-alpha-range simulated scalp EEG resulting from all dipole source activity (visual and background dipoles). Data points are generated from the seven different interdipole dependency models and are ordered form left to right in terms of increasing dependency/integration (full independency model, independent model—level 2, independent model—level 1, interdependent model—level 3, interdependent model—level 2, interdependent model—level 1, full dependency model). Red lines, high amplitude simulations; black lines, low amplitude simulations. Data points reflect averages across 22 separate simulations; standard errors of simulated C_I_(X) and I(X) are too small to be displayed but range from 0.04 to 0.15 bits.

**Figure 9:**
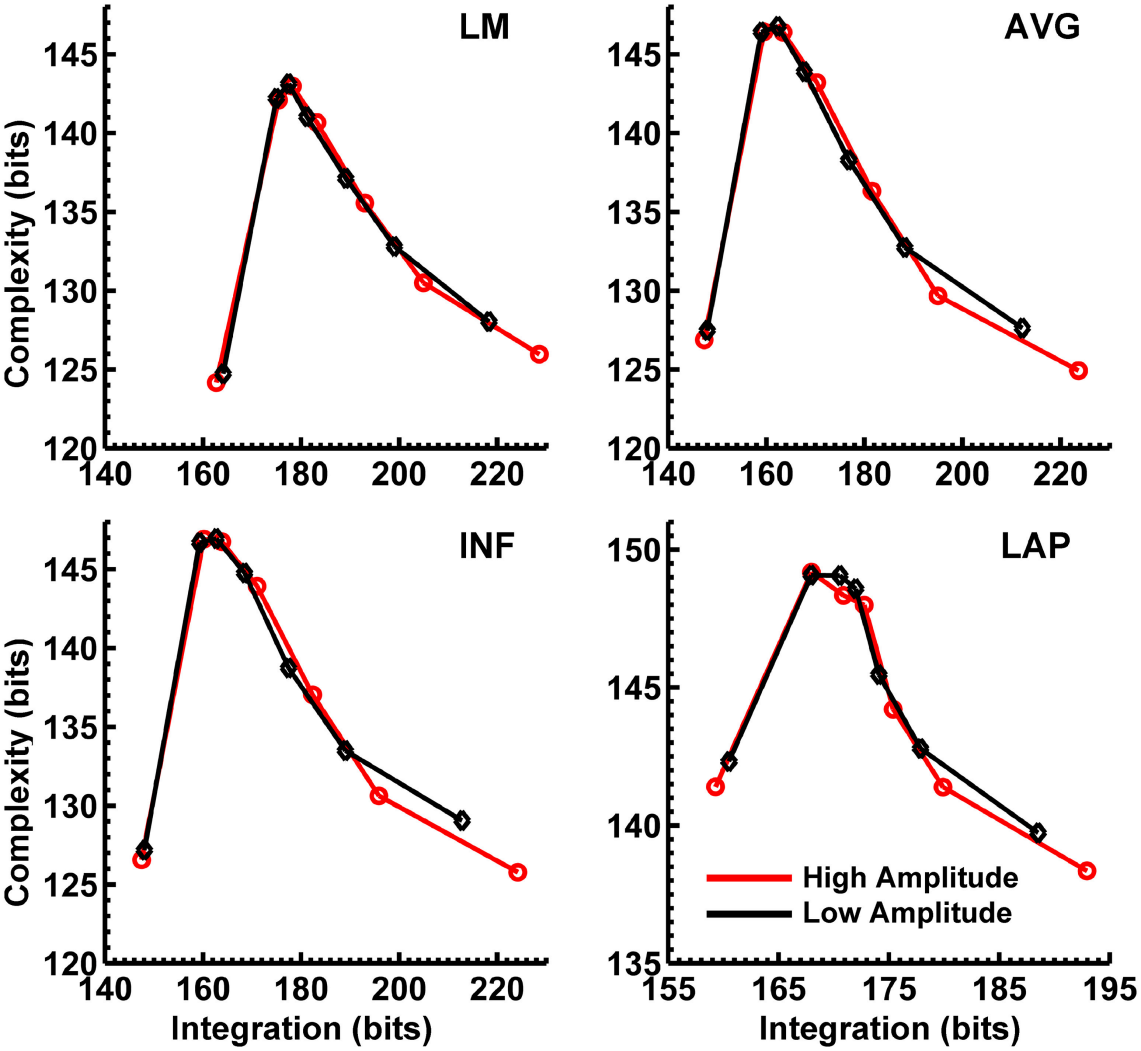
Interaction complexity C_I_(X) as a function of integration I(X) for beta-range simulated scalp EEG resulting from all dipole source activity (visual and background dipoles). Data points are generated from the seven different interdipole dependency models and are ordered from left to right in terms of increasing dependency/integration (full independency model, independent model—level 2, independent model—level 1, interdependent model—level 3, interdependent model—level 2, interdependent model—level 1, full dependency model). Red lines, high amplitude simulations; black lines, low amplitude simulations. Data points reflect averages across 22 separate simulations; standard errors of simulated C_I_(X) and I(X) are too small to be displayed but range from 0.04 to 0.40 bits.

Figures 8, 9 display mean C_I_(X) as a function of mean I(X) for the theta-alpha-range and beta-range simulated scalp EEG resulting from volume-conduction of all simulated dipole source activity (extrastriate and background dipoles). The figures show results for the simulated data reference with respect to all four EEG references. In the high integration range where theoretically C_I_(X) is a monotonically decreasing function of I(X) (see Figure 1), all four EEG references were able to correctly reproduce the gradient of the complexity-integration relationship among the visual dipole moments. However, in the low integration range, where theoretically C_I_(X) is a monotonically increasing function of I(X), the functional C_I_(X) vs. I(X) pattern is distorted from that seen for the dipole moment sources for all four EEG references. Although the same basic inverted-U pattern is present at the scalp level, C_I_(X) reaches a maximum for the level-2 independent model, rather than the level-1 independent model in the low integration range. This distortion was also present when the background sources were removed from the simulation and scalp level signals were generated from the extrastriate sources only (see Figures S1, S2). In addition, scalp-level C_I_(X) was smaller for the high amplitude vs. the low amplitude extrastriate dipole moments in the context of the background noise sources^1^. This C_I_(X) pattern is similar to that seen for the observed eyes closed vs. open EEG resting states, but opposite that seen for C_I_(X) computed from the simulated dipole sources directly. However, this distortion was absent when the background sources were removed from the simulations (Figures S1, S2); then C_I_(X) was larger for high vs. low amplitude simulations, in agreement with the C_I_(X) pattern computed from the dipole source moments directly. Possible reasons for these differences between the full dipole model (extrastriate + background dipoles) and the extrastriate dipole only models are taken up in the Discussion section, below.

Finally, Figures 8, 9 show that scalp-level complexity was largest, and scalp-level integration was smallest, for the simulated LAP-referenced data. This is the same pattern observed for the LAP-referenced empirical data. In addition, comparison of Figures 8, 9 with Figure 7 indicate that the absolute complexity and integration values at the scalp are much higher than those observed for the dipole sources, an inflation most likely due to volume conduction (see also Tables S3, S4).

## Discussion

The present study investigated the effect of four EEG reference schemes on the quantification of EEG complexity C_I_(X) and integration I(X) during a resting state task. In the context of a simple measurement of resting state EEG, we found that EEG reference influenced both the magnitude and sensitivity to volume-conduction artifacts of scalp EEG C_I_(X) and/or I(X) collapsed across eyes open and eyes closed resting state conditions, as well as the magnitude of scalp EEG C_I_(X) and I(X) differences between resting state conditions. For all EEG references, these effects were observed in the context of theta/alpha-range and beta-range PSD differences previously reported for resting state EEG recordings (i.e., greater PSD for eyes closed vs. eyes open resting states; Kornguth et al., 2013; Witkowski et al., 2015). However, consistent with previous reports (e.g., Yao et al., 2005), the magnitude of these spectral power differences also varied according to EEG reference. In addition, we performed dipole source modeling in order to assess the accuracy of scalp-level estimates of complexity and integration of neural sources and how these estimates are affected by EEG reference choice. The dipole source modeling showed that simulated scalp EEG-level C_I_(X) and I(X) accurately reflect changes in underlying neural source dependencies when using all four EEG references, but only in the high integration range.

### Effects of EEG reference on scalp EEG complexity

Our first main finding was that scalp EEG interaction complexity C_I_(X) was largest for the LAP transformation, smallest for the LM reference, and at intermediate values for INF and AVG references for both theta-alpha and beta frequency ranges. However, the surrogate data analysis indicated a differential across-reference pattern of non-random or non-spuriously coincident complexity arising due to volume conduction. In the theta-alpha frequency range, the C_I_(X) values of both resting states lay outside the surrogate confidence intervals for the LAP, INF, and AVG EEG references, whereas LM-referenced data were outside the surrogate confidence intervals only for the eyes closed data. In the beta frequency range, only the C_I_(X) values of both resting states for the LAP reference lay outside the surrogate confidence intervals. Thus, of all the EEG references, the LAP reference was the most “robust” in the sense that it returned large-magnitude C_I_(X) values that were less sensitive to volume-conduction artifacts across both EEG frequency ranges and resting state conditions. We suggest that this pattern reflects two characteristics of an EEG reference: (1) its impact on neuroelectric signal quality and statistics, and (2) the accuracy with which neuroelectric signals measured with respect to a particular reference can represent the activity of the cortical sources underlying EEG topographies. LAP-transformed EEG signals possess higher levels of both characteristics relative to the other reference schemes. First, these signals are “reference free” estimates of radial (transcranial) current flow entering and leaving the scalp, and thus are not prone to across-electrode contamination of activity from a single physical scalp location, such as a monopolar recording reference site. Second, the LAP-transformed signals provide an enhanced representation of superficial EEG current generators that are (mostly) radially-oriented, at the expense of less sensitivity to deep sources and/or spatially-broad activities arising from distributed sources (Pernier et al., 1988; Law et al., 1993; Dien, 1998; Kayser and Tenke, 2006a,b). However, we argue that the last characteristic may actually be beneficial for the assessment of EEG complexity because some information about those distributed sources is likely lost in the constructive summation of their activity that produces the spatially-broad EEG response across the scalp. Instead, the LAP transformation distinguishes the local activity of those distributed sources from each other in a manner that is highly informative. Evidence that the LAP data provided a more robust measurement of EEG source activity than the other references is given by the present observation that LAP-transformed signals had the highest PSD values of the four different EEG reference schemes. This finding was not due to the fact that the LAP-transformed PSD values are measured in different units (μV^2^/m^4^/Hz) than the other references (μV^2^/Hz), because all PSD values were converted to decibel units before statistical comparison (see Computation of Resting EEG Power Spectral Density section, above). Moreover, the GEE-based regression analysis indicated that C_I_(X) level was positively associated with PSD magnitude across EEG references schemes, again suggesting that the EEG reference scheme that provides a more accurate assessment of EEG source activity also provides a robust estimate of EEG complexity.

Taken together, the above findings support the conclusion that the LAP-transformed EEG data provides the most robust estimates of EEG complexity relative to the other EEG reference schemes. In contrast to the LAP transformation, the LM reference, being close to the jaw and neck, is fairly noisy and highly sensitive to subtle head/neck movements and muscle activity. Moreover, LM-referenced EEG signals express changes in transverse scalp electric potential rather than radial current, and thus provide a spatially-limited representation of the underlying cortical sources. Hence, as we observed, the LM reference should provide the least robust estimate of EEG complexity. The AVG and INF references also provide spatially-limited representations of cortical EEG sources. However, given that these references approximate a noiseless zero potential with sufficient spread and density of electrode coverage across the scalp (Bertrand et al., 1985; Yao, 2001), they should produce more informative, and thus complex, EEG signals than the LM reference, again as we observed.

We note that the present observation of larger complexity for the LAP transformation is in apparent contrast to the observation of van Putten and Stam (2001), who found the magnitude of a related measure of EEG complexity [neural complexity C_*N*_(X)] to be larger for an average reference vs. a source reference consisting of the voltage difference between a given recording site and the mean voltage of 3–4 neighboring recording sites. As described, this source reference appears to be a multi-channel version of a bipolar montage with the EEG signals still expressed in terms of transverse scalp electric potentials rather than radial scalp current flow. Thus, it is unclear to what degree this reference can spatially represent cortical EEG sources, how susceptible it is to local noise, and the effects this would have on the computation of EEG complexity relative to the LAP-transformation. This should be a subject of further research.

### Effects of EEG reference on scalp EEG integration

The second main finding of the present study is that EEG integration I(X) was smallest for the LAP transformation, largest for the LM reference, and at intermediate values for the INF and AVG references for both theta-alpha and beta frequency ranges. Surrogate data analysis showed that computed I(X) values were unlikely to be due to random or spuriously coincident volume-conducted EEG activity for any of the EEG references. The GEE-based regression analyses indicated that this change in I(X) was accompanied by an opposing change in PSD across EEG reference schemes, with larger EEG integration values associated with lower EEG power and vice versa. Integration is a measure of the overall deviation from statistical independence of the individual elements of a multivariate system. Thus, I(X) should be affected by factors that influence the measured independence of a set of signals. Volume conduction is one such factor because volume-conducted signals may be detected at neighboring scalp channels and thus can introduce a non-physiological source of correlation between their measured signals. Given that the LAP transformation reduces the effects of volume conduction and is reference free (Law et al., 1993; Kayser and Tenke, 2006a,b; Nunez and Srinivasan, 2006), integration should be smallest for this reference, as we observed. This observation is consistent with the report of van Putten and Stam (2001) of a lower overall level of integration for the source reference vs. the average reference. On the other hand, derivation of the EEG signals relative to LM, AVG, and INF references should have no effect on volume conduction. Moreover, these monopolar references may cause activity at reference sites to be shared across the other EEG channels and thus introduce a form of artifactual correlation between them. These effects should be greatest for the LM reference given its proximity to the neck, jaw, and lower head muscles. The AVG and INF references are less prone to the correlating effects of common reference activity because most of the idiosyncratic activity of individual EEG channels is averaged out in the construction of the AVG reference (unless a large number of channels show high-amplitude, synchronous activity), whereas the INF reference estimates a neutral (noiseless) reference at infinity. Hence, though all four EEG references will express true and artifactual integration among EEG signals, the LM reference should produce larger integration values than the AVG and INF references, which in turn should be larger than the LAP transformation, as we observed.

### The interaction of EEG statistics and EEG reference on EEG complexity and integration

Although the present EEG integration findings are consistent with previous reports (van Putten and Stam, 2001; van Cappellen van Walsum et al., 2003; Rapp et al., 2005), we note that the direction of between-resting state condition differences in complexity is not. These previous studies found a reduction in complexity during the eyes open vs. eyes closed resting state conditions (van Putten and Stam, 2001; van Cappellen van Walsum et al., 2003; Rapp et al., 2005), in contrast to the present observations for C_I_(X). What might account for this discrepancy between past studies and ours? van Putten and Stam (2001) suggested their observations may be due to use of a low-spatial resolution scalp montage (21 channels) and that the mixture of different signal frequencies in the electroencephalogram decrease interelectrode correlations in wideband data even though the electrodes are mostly synchronized within a single frequency range. We bandpass-filtered our data in narrow ranges and used a high-density scalp montage (72 channels) to rule out these concerns. However, other studies have observed the same resting state condition differences as van Putten and Stam (2001) in filtered data with a high-density MEG montage (van Cappellen van Walsum et al., 2003).

Instead, we suggest this discrepancy between our study and previous reports may arise from the fact that the analytic expressions for the complexity and integration measures utilized in all of these studies assume the EEG signals to be approximately Gaussian distributed (Norwich, 1993; Tononi et al., 1994; van Putten and Stam, 2001; Ince et al., 2017). It is well known that this assumption is often partially or completely violated for physiological signals (Dumermuth, 1968; Elul, 1969; Dumermuth et al., 1972; Pollock et al., 1990). In the present study, our EEG data exhibited a substantial deviation from normality (see Methods—Computation of EEG Complexity and Integration section). Thus, we applied a method to transform non-normal distributions to Gaussian that has been successfully used before with EEG data (van Albada and Robinson, 2007). We then verified that the transformed data met univariate and multivariate Gaussian assumptions before computing C_I_(X) and I(X) (see Methods—Computation of EEG Complexity and Integration section). Additionally, we carefully verified that the Gaussian transformation did not distort key resting state EEG features (see Supplementary Materials). However, to our knowledge, the previous studies observing greater complexity for eyes closed than eyes open resting state conditions assumed a Gaussian distribution for their data, but did not report any assessment of how well this assumption fit their data sets. Hence, if the physiological data of these previous studies either partially or completely failed to meet Gaussian statistical assumptions, then this might explain the discrepancy between past studies and ours regarding resting state complexity differences. It is entirely possible that the character of EEG or MEG statistics departs from normality to a greater degree or lesser degree in one resting state condition relative to the other, which would produce inaccurate estimations of C_I_(X) and I(X).

To test this hypothesis, we computed C_I_(X) and I(X) on the original non-Gaussian-transformed data (see Supplementary Material) and found resting state C_I_(X) to be greater for the eyes closed than eyes open resting state condition, which was the pattern reported by previous reports (van Putten and Stam, 2001; van Cappellen van Walsum et al., 2003; Rapp et al., 2005). The overall resting state integration pattern was unchanged by the Gaussian transformation, however, and was also consistent with previous results. Importantly, these earlier studies used several seconds' worth of data to compute C_I_(X) and I(X), whereas here we used 1-s (256-sample) trials (see Methods—EEG Recording and Pre-Processing). The similarity between previous observations and the present non-Gaussian-transformed results rule out the possibility that our use of short trials may have biased the statistical distribution of the data toward non-normality, or were otherwise too short to provide a correct estimate of the statistical properties of the data, in a manner that affected the computation of EEG complexity. Moreover, our statistical testing indicated that, at the very least, the Gaussian-transformed data better met the required statistical assumptions of the entropy formulas then the non-transformed data (see Methods—Computation of EEG Complexity and Integration).

Hence, we conclude that the computation of C_I_(X) via the analytical expressions used in the present and past studies is highly dependent on the degree to which the data meet the Gaussian statistical assumptions. One should always employ a verification and/or correction procedure such as the one we utilized in this study. Alternatively, one may utilize discrete methods of computing EEG entropy that do not require data to be distributed in a particular way (Misra et al., 2005; Magri et al., 2009). This matter is relevant to the main issue of EEG reference choice explored in this paper, because different reference transformations may change the statistics of the EEG signals in various ways, which in turn may affect the computation of C_I_(X) and I(X) across references. In fact, we did find different across-reference patterns of C_I_(X) and I(X) for the non-Gaussian-transformed data relative to the transformed data (see Supplementary Material).

### Scalp-level estimation of neural source complexity and integration

We performed dipole source modeling-based simulations in order to assess the accuracy of scalp-level estimates of complexity and integration of neural sources and how these estimates are affected by EEG reference choice. Our simulations were based on a 4-shell spherical model (Cuffin and Cohen, 1979; Mosher et al., 1993; Tenke and Kayser, 2015) with 40 oscillating dipole sources spread over posterior extrastriate cortical shell regions, and 148 oscillating background “noise” dipoles equally spread over the remainder of the cortical shell. We created different statistical dependencies among the extrastriate dipoles in order to recreate different points of the theoretical “inverted-U” non-monotonic complexity-integration function (Tononi et al., 1994; see Figure 1) at the neural source level. We then computed the forward solutions of these source configurations, referenced the simulated scalp EEG with respect to all four EEG references examined here, and computed complexity and integration.

Our simulations reproduced the basic scalp topography of between-resting state condition differences in PSD (Figure 6). We also found that the dipole sources followed the theoretical complexity-integration curve (Figure 7), with fully independent sources yielding low complexity and integration values, fully dependent sources yielding low complexity and high integration, and heterogeneously dependent sources yielding high complexity and intermediate integration values. At the level of simulated scalp EEG (Figures 8, 9), C_I_(X) and I(X) correctly reproduced the gradient of the complexity-integration relationship among the extrastriate dipole sources in the high integration range where C_I_(X) is a monotonically decreasing function of I(X). This was the case for all four EEG references. However, in the low integration range—where dipole C_I_(X) is a monotonically increasing function of I(X)—the scalp-level pattern of C_I_(X) and I(X) was distorted from that seen for the dipole moment sources for all four EEG references. Here, scalp-level C_I_(X) reached a maximum at the level-2 stage of independence among the dipoles, rather than the level-1 independence stage as observed for C_I_(X) computed from the dipole sources directly. Interestingly, this distortion was also present if the background sources were removed from the simulation (see Figures S1, S2), suggesting that it results from the mixing effects of the extrastriate source signals due to volume conduction. This scalp-level distortion of the complexity-integration function of the neural sources may represent a fundamental limitation of the use of scalp-recorded EEG to estimate neural source complexity in the low integration range. However, this limit may not pose a problem for most EEG studies, as the neural processes detectable by EEG are oscillatory in nature and thus likely operate at higher levels of integration. This is because oscillating signals, even those with random phases, have an intrinsic non-random autocorrelative structure that cannot be broken down further without changing the periodicity of the signals. In the absolute limit, full randomization would cause these signals to either become non-oscillatory or turn into white noise with equal spectral power across frequencies. In this case, scalp signals would either no longer be detectable as rapidly alternating voltage fluctuations (although they may be present as slow DC potentials) or if detectable, would manifest as very low levels of complexity and integration resolvable at the scalp (for example, as seen for the fully independent dipole source model).

Our simulations also yielded smaller scalp-level C_I_(X) for the high vs. low amplitude extrastriate dipole sources at intermediate and high levels of integration (Figures 7, 8). This is a pattern similar to that observed for the empirical eyes closed vs. open EEG resting state data. However, this scalp-level C_I_(X) difference pattern was reversed when the background sources were removed from the simulations (Figures S1, S2). Here, C_I_(X) was larger for high vs. low amplitude simulations, in agreement with the C_I_(X) pattern computed from the dipole source moments directly. This was the case for all four EEG references. We suggest that the reversed scalp-level C_I_(X) differences seen in the full dipole model results from the volume conduction of the background noise sources in the model. The high level of scalp complexity that arises from the large number of randomly oscillating background sources likely dominated the output of the C_I_(X) estimator, with this dominance modulated by the presence of the partially- or fully-dependent extrastriate sources. As the latter increased in amplitude, they contributed more to the scalp signal, thus reducing scalp-level complexity and increasing scalp-level integration.

We note that the absolute values of C_I_(X) and I(X) were considerably higher than the corresponding values for the dipole sources; this is likely due in part to the added complexity of the additional background dipole sources. However, volume conduction also played a role in the inflation of C_I_(X) and I(X) values at the scalp because this inflation was also observed when the background sources were removed from the simulation and scalp level signals were generated from the extrastriate sources only (see Figure S1 and Tables S3, S4). Nevertheless, regardless of whether the background sources were present or not in the simulation, scalp-level C_I_(X) values were highest, and I(X) values were lowest, for the simulated Laplacian-transformed data, as observed for the empirical EEG data. These observations suggest that the LAP reference produces the closest approximation to the true absolute dipole source integration values, but the worst approximation to the true absolute source complexity values. However, researchers are more often interested in between-source dependency level differences across different EEG references and experimental conditions. In this case, a better criterion for EEG reference performance is a source- vs. scalp-level comparison between complexity and integration gradients across source dependency levels. We compared these gradients for the case when the background sources were removed from the simulation and scalp level signals were generated from the extrastriate sources only (see Table S5). This analysis showed that, with the exception of theta-alpha-range integration, the Laplacian-referenced data was better able to reproduce the gradients of complexity and integration changes across source dependency levels than the other EEG references.

**Figure 10:**
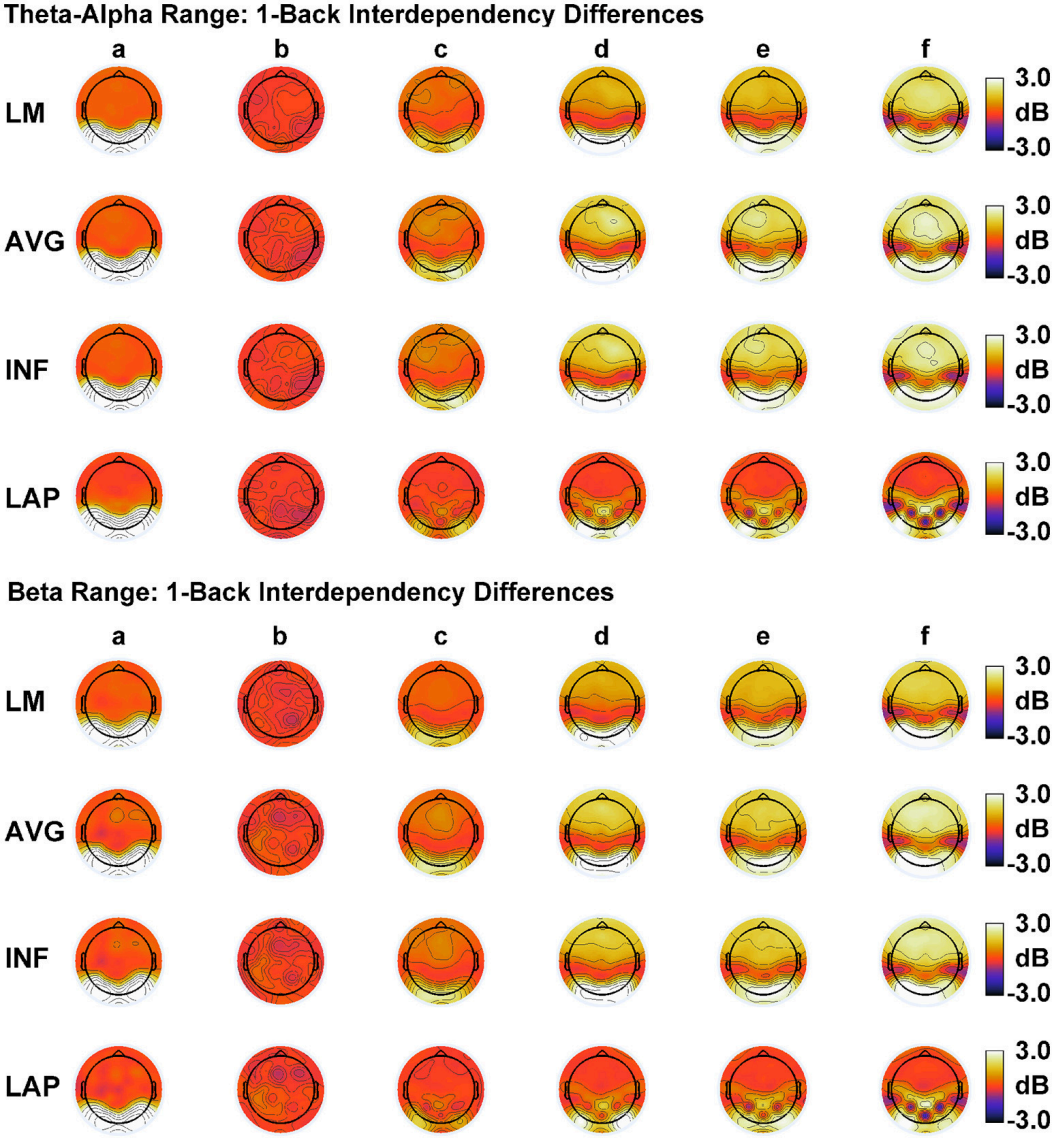
Resting state EEG PSD differences may result from differences in dipole source dependencies. Representative pairwise interdependency level *n* + *1*—level *n* (“1-back”) scalp PSD topography differences for simulated theta-alpha range **(top)** and beta-range **(bottom)** EEG data. **(a)** independent model-level 2—full independency model, **(b)** independent model-level 1—independent model-level 2, **(c)** interdependent model-level 3—independent model-level 1, **(d)** interdependent model-level 2—interdependent model-level 3, **(e)** interdependent model-level 1—interdependent model-level 2, **(f)** full dependency model—interdependent model-level 1. Head maps display PSD topographies averaged over the stated frequency ranges; light/dark colors indicate ± values. Scalp maps reflect averages across 22 separate simulations.

Finally, we note that the observed C_I_(X) was larger, and observed I(X) was smaller, for the eyes open vs. eyes closed resting state conditions, regardless of choice of EEG reference scheme. The GEE-based regressions showed that C_I_(X) was negatively related, and I(X) was positively-related, to PSD magnitude across resting state conditions. It is unclear from our simulations if these PSD, C_I_(X), and I(X) differences reflect passive volume-conducted differences in neural source amplitudes between resting state conditions and the resultant differences in interelectrode correlations at the scalp, or if they reflect between-condition differences in the dependency relationships among the neural sources (irrespective of differences in neural source amplitude). The latter case could produce between-condition differences in EEG power without an increase in the amplitude of individual EEG sources. This is illustrated in Figure 10, which shows example mean PSD differences between successive interdipole-dependency levels for the high amplitude simulations. (Other interdipole dependency levels contrasts are possible, such as the differences between *n*+*2* and *n* dependency levels.) The figure shows that simulations with high I(X) and low C_I_(X) produced higher PSD levels than simulations with lower I(X) and C_I_(X) levels. This explanation would be consistent with evidence that during the eyes closed resting state the visual cortex is driven by endogenous pacemakers in the thalamus which synchronize much of the visual cortex in the alpha, theta and beta ranges (Adrian and Yamagiwa, 1935; Buzáki, 1992). This explanation would also be consistent with the hypothesis that in order to perform specialized information-processing during rich neurocognitive conditions (such as the eyes open resting state), the brain organizes itself into distributed neuronal groups that interact more strongly with themselves than with the rest of the brain (Tononi et al., 1994, 1996, 1998a). Determining which neural source model best explains the observed resting state data could be achieved by either (1) fitting the empirical data to a dipole model with free parameters (dipole source frequency, phase, amplitude, and interdipole dependencies) that minimize an objective error function, or (2) conducting a source localization analysis (e.g., beamformer, minimum norm estimation, LORETA) and quantifying the complexity and integration of the localized sources. These analyses are beyond the scope of the present study and are a subject for future research.

### Study limitations

We performed dipole simulations that provided a benchmark to compare the ability of scalp-level C_I_(X) and I(X) measures to resolve the complexity and integration of neural sources and how these estimates are affected by choice of EEG reference. Nevertheless, our models specifically described resting state conditions that involved ongoing non-phase-locked EEG oscillations with varying statistical dependencies among posterior cortical sources. Our models also did not examine situations in which the number and location of dependent sources varied between conditions. Moreover, our models were phenomenological in the sense that they did not directly simulate the interactions through which neural groups form interdependent relationships. That said, we believe our findings will generalize to other tasks that engage similar ongoing non-phase-locked EEG activity (such as mental arithmetic, motor grasping, or vigilant attention tasks). However, they may not generalize to tasks that engage different kinds of neural dynamics, such as evoked potentials time-locked to specific events, or conditions that engage vastly different mental states (e.g., sleep, anesthesia). Moreover, our study utilized a 72-channel sensor montage; it is unclear how the number of channels affects the computation of C_I_(X) and I(X). Future research is needed to determine how these factors affect the quantification of C_I_(X) and I(X), and how this interacts with the choice of EEG reference. One guiding principle that should assist such endeavors is the theoretical complexity-integration function (Figure 1), which is a general relationship that should apply across all experimental situations.

## Conclusion

In conclusion, we have shown that information-theoretic measures of integration I(X) are relatively robust to volume-conduction artifacts across all four EEG references when comparing resting state condition differences, whereas complexity C_I_(X) is the most robust to volume-conduction artifacts when computed with respect to the LAP reference. In addition, dipole simulations showed that of the four EEG references, the LAP reference produced the closest approximation to the true absolute dipole source integration values, and more accurately estimated the gradients of complexity and integration changes across source dependency levels. Moreover, the magnitude of the resting state C_I_(X) and I(X) differences were generally lowest for the LAP transformation, suggesting that LAP-transformed data provides a conservative estimate of between-condition complexity and integration differences. Thus, when measuring EEG complexity and integration during resting states (or similar tasks that involve ongoing, relatively stationary EEG signals), we recommend use of the Laplacian-transformation due to its positive impact on EEG signal quality, sharpening of source topography, reduction of volume-conduction effects, and the resultant positive effect these have on the measurement of complexity and integration. Although average or infinity references do not reduce volume conduction, their use for the computation of EEG complexity and integration is acceptable in situations when the Laplacian-transformation is precluded (i.e., the expectation of deep cortical sources) and when they can approximate a neutral reference (i.e., when there is sufficient spread and density of electrode coverage across the scalp; Junghöfer et al., 1999; Liu et al., 2015). Furthermore, although we found the average and infinity references to display roughly equivalent performance for the computation of complexity and integration, the infinity reference is to be favored due to its greater accuracy in representing resting state EEG activity (Qin et al., 2010). Finally, we do not recommend use of a linked-mastoid reference for the computation of EEG complexity and integration due to its greater noise levels and tendency to induce artifactual correlations among scalp electrodes.

## Author contributions

LT contributed to the experimental design, data collection and analysis, and manuscript preparation. CS and RV contributed to the data collection and analysis, and manuscript preparation.

### Conflict of interest statement

The authors declare that the research was conducted in the absence of any commercial or financial relationships that could be construed as a potential conflict of interest.

## Footnotes

^1^We also performed simulations with a 400% amplitude difference in order to investigate the effects of amplitude on EEG complexity and integration (although such an amplitude difference is unlikely to be biologically typical of the two resting state conditions). This increased scalp-level integration in the full dependency condition by about 35–40 bits depending on EEG reference, with the other dependency conditions unaffected. We believe this pattern obtains because complexity and integration are differential entropies (i.e., they involve the subtraction of two entropy terms; see Methods, Computation of EEG Complexity and Integration section) that are independent of scale (except for signal dispersion effects due to volume conduction, which are likely greatest in the full dependency condition).
