# Supplementary material for: The Effect of Electroencephalogram (EEG) Reference Choice on Information-Theoretic Measures of the Complexity and Integration of EEG Signals
Source: Front Neurosci. 2017 Jul 25;11:425. doi: 10.3389/fnins.2017.00425 (PMC5524886; doi:10.3389/fnins.2017.00425)
Supplement: Supplementary file 1 [file DataSheet1.pdf]

Supplementary Material

The effect of electroencephalogram (EEG) reference choice on information-theoretic measures of the complexity and integration of EEG signals

Logan T. Trujillo*, Candice T. Stanfield, & Ruben D. Vela

*** Correspondence:** Corresponding Author: logant@txstate.edu

1 Supplementary Data:

Effects of Gaussian Transformation on EEG Features. Here we report that Gaussian-transformation of our EEG data did not distort the typical EEG features of the eyes open and closed resting states. First, we compared the single-trial distributions of EEG PSD and signal phase between the non-Gaussian-transformed and Gaussian-transformed data separately for the eyes open and eyes closed conditions. Single-trial power and phase values were extracted via FFT for each participant and individual frequency within the theta-alpha and beta bands. Then we performed nonparametric two-sample Kolmogorov-Smirnov (KS) tests of the equality of power and phase distributions at the $p < .05$ two-tailed level, with multiple comparisons across electrodes and frequencies corrected via the Holm-Bonferroni procedure (Holm, 1979). KS tests are ideal for this purpose, as they are sensitive to distributional differences in both location and shape (Massey, 1951). The KS tests were performed separately for each participant, electrode, individual frequency within a frequency band, resting state condition, and EEG reference.

None of the KS tests were significant for the theta-alpha band data. For the beta-range data, two participants exhibited significant power and phase differences over 8 – 15 electrode-frequency points only for the LM EEG reference. These differences reflected slight EEG power decreases and small phase changes at these electrode-frequency points between the non-Gaussian-transformed versus Gaussian-transformed data in both the eyes open and closed conditions. However, these differences were very small for these participants (beta power: ~ 0.5% change on average; beta phase: ~ 4% change on average) and likely produced a negligible distortion of their beta EEG signals. In general, the overall EEG theta/alpha and beta PSD and phase values at each electrode site after Gaussian transformation were very close to the values of the non-transformed data across participants. Importantly, the overall between-resting state condition pattern of ANOVA results for the Gaussian-transformed EEG PSD (see Table S1, below) was the same as for the untransformed data reported in the main manuscript (Table 1).

We also assessed what, if any, changes the Gaussian-transformation may have produced in the relative phases among recording electrodes; relative phase is an EEG feature that likely has a substantial impact on complexity and integration measures. We assessed this by computing the inter-electrode phase synchrony for all possible electrode pairs and comparing this between the Gaussian-transformed and non-transformed data. Phase synchrony was computed for each individual trial using a special statistic designed to assess the average amount of phase-locking between electrode pairs within small time windows of a single trial (single-trial phase-locking value, or S-PLV; Lachaux et al., 2000). Here, we used the S-PLV statistic to assess phase synchrony across the entire single-trial

time range. The S-PLV statistics were computed from the phase angles extracted from theta-alpha-filtered and beta-filtered data via the MATLAB *angle* function. (Note that the phase angles were extracted from the composite band-passed signals, rather than on the individual frequencies within a frequency band, because the composite band-passed signal is what was entered into the EEG complexity and integration calculations.) We then performed the two-sample KS tests for the equality of S-PLVs separately for each participant, electrode pair, frequency band, resting state condition, and EEG reference. None of the KS tests were significant, suggesting that the Gaussian transformation did not overly distort the relative phases of the EEG between electrodes.

EEG Band	EEG Reference	Eyes Closed	Eyes Open	EEG Band	EEG Reference	Eyes Closed	Eyes Open
Theta/ Alpha	LM	24.85 (0.60)	20.36 (0.53)	*Beta*	LM	15.70 (0.52)	13.74 (0.46)
	AVG	24.31 (0.63)	19.41 (0.54)		AVG	14.51 (0.50)	12.17 (0.39)
	INF	24.12 (0.64)	19.22 (0.55)		INF	14.45 (0.50)	12.07 (0.39)
	LAP	40.82 (0.70)	35.61 (0.62)		LAP	31.51 (0.56)	29.10 (0.49)

Table S1. Mean Gaussian-transformed EEG power spectral density by EEG frequency band, resting state condition, and EEG reference. All values are in dB; SE in parentheses.

Complexity and Integration of Non-Gaussian-Transformed EEG Data. Next, we report the results of computing $C_I(X)$ and $I(X)$ values on our EEG data prior to Gaussian-transformation (Table S2). We found that, contra our Gaussian-transformed results (see main manuscript) and in agreement with previous studies, $C_I(X)$ was larger for the theta/alpha-range eyes closed versus eyes open condition (272.72 ± 1.91 bits versus 266.70 ± 1.78 bits). No significant differences were found for the beta-range $C_I(X)$ comparison, (337.48 ± 2.66 bits versus 336.76 ± 2.57 bits). However, in agreement with previous studies and the Gaussian-transformed analysis, $I(X)$ was larger for the eyes closed versus eyes open condition in both frequency ranges (Theta/alpha-range: 497.69 ± 4.33 bits versus 469.22 ± 3.54 bits; Beta-range: 338.81 ± 2.80 bits versus 328.33 ± 2.57 bits).

We also observed a different pattern of $C_I(X)$ and $I(X)$ differences across EEG references for the non-Gaussian data relative to the Gaussian-transformed data reported in the main manuscript. Interaction complexity was largest for the LAP reference (Theta/alpha-range: 338.88 ± 1.91 bits; Beta-range: 425.57 ± 2.72 bits), followed by the LM reference (Theta/alpha-range: 248.32 ± 1.84 bits; Beta-range: 309.41 ± 2.55 bits), the INF reference (Theta/alpha-range: 246.34 ± 1.78 bits; ; Beta-range: 307.31 ± 2.52 bits), and the AVG reference (Theta/alpha-range: 245.29 ± 1.79 bits; ; Beta-range: 306.12 ± 2.53 bits). Integration followed a similar across-reference order as $C_I(X)$, being largest for the LAP reference (Theta/alpha-range: 531.84 ± 3.06 bits; Beta-range:371.29 ± 2.69 bits), followed by the LM reference (Theta/alpha-range: 487.14 ± 4.15 bits; Beta-range: 341.51 ± 3.28 bits), the INF reference (Theta/alpha-range: 459.29 ± 4.03 bits; Beta-range: 312.19 ± 2.84 bits), and the AVG reference (Theta/alpha-range: 455.55 ± 4.14 bits; Beta-range:309.28 ± 3.00 bits). These findings suggest that non-normal distribution of the EEG signals can profoundly distort estimates of

complexity and integration when computed using analytical expressions for $C_I(X)$ and $I(X)$ (see main text for further discussion).

EEG Band	EEG Measure	Effect	F	p	ε	η^2_P
Theta/Alpha	$C_I(X)$	*REF*	126532.66	†.001	.58	.99
		RS	132.68	.001	–	.86
		REF x RS	45.32	†.001	.63	.68
	$I(X)$	*REF*	804.10	†.001	.67	.98
		RS	114.40	.001	–	.85
		REF x RS	69.67	†.001	.55	.77
Beta	$C_I(X)$	*REF*	72894.87	†.001	.42	.99
		RS	0.671	.422	–	.03
		REF x RS	0.441	†.573	.46	.02
	$I(X)$	*REF*	295.11	†.001	.59	.93
		RS	44.12	.001	–	.68
		REF x RS	79.77	†.001	.56	.79

Table S2. Analysis of variance (ANOVA) results for EEG interaction complexity $C_I(X)$ and integration I(X) for each frequency band with Gaussian-transformation of the EEG data (see Material and Methods - Computation of EEG Complexity and Integration section of the main manuscript). ANOVA factor labels: REF, EEG Reference; RS, EEG Resting State. REF factor effects *dfs* = 3, 63; RS main effect *df* = 1, 21. The † symbol indicates *p* values subject to Greenhouse-Geisser correction (see Materials and Methods – Statistical Analysis of EEG/ERP Measures section).

Extrastriate-Only Dipole Simulations. Here we report the $C_I(X)$ and I(X) of the scalp-level EEG that arises when only the posterior extrastriate dipole sources are included in the source model. Figures S1 and S2 show scalp-level mean values for the theta-alpha band and beta band extrastriate sources, respectively. Note that the fully independent dipoles were formed from multivariate Gaussian white noise that was then mixed with theta-alpha or beta band background sources in the full dipole model; thus fully independent $C_I(X)$ and I(X) are the same for the theta-alpha and beta range simulations. These figures show that for the extrastriate-only dipole simulations, in contradistinction to the full (extrastriate and background) dipole model, 1) high amplitude simulations tended to produce greater $C_{I,}(X)$ values than the low amplitude simulations and 2) scalp-level $C_I(X)$ reached a maximum at level-1 interdipole independency. In contrast, the basic scalp-level EEG integration patterns were similar between the extrastriate-only and full models.

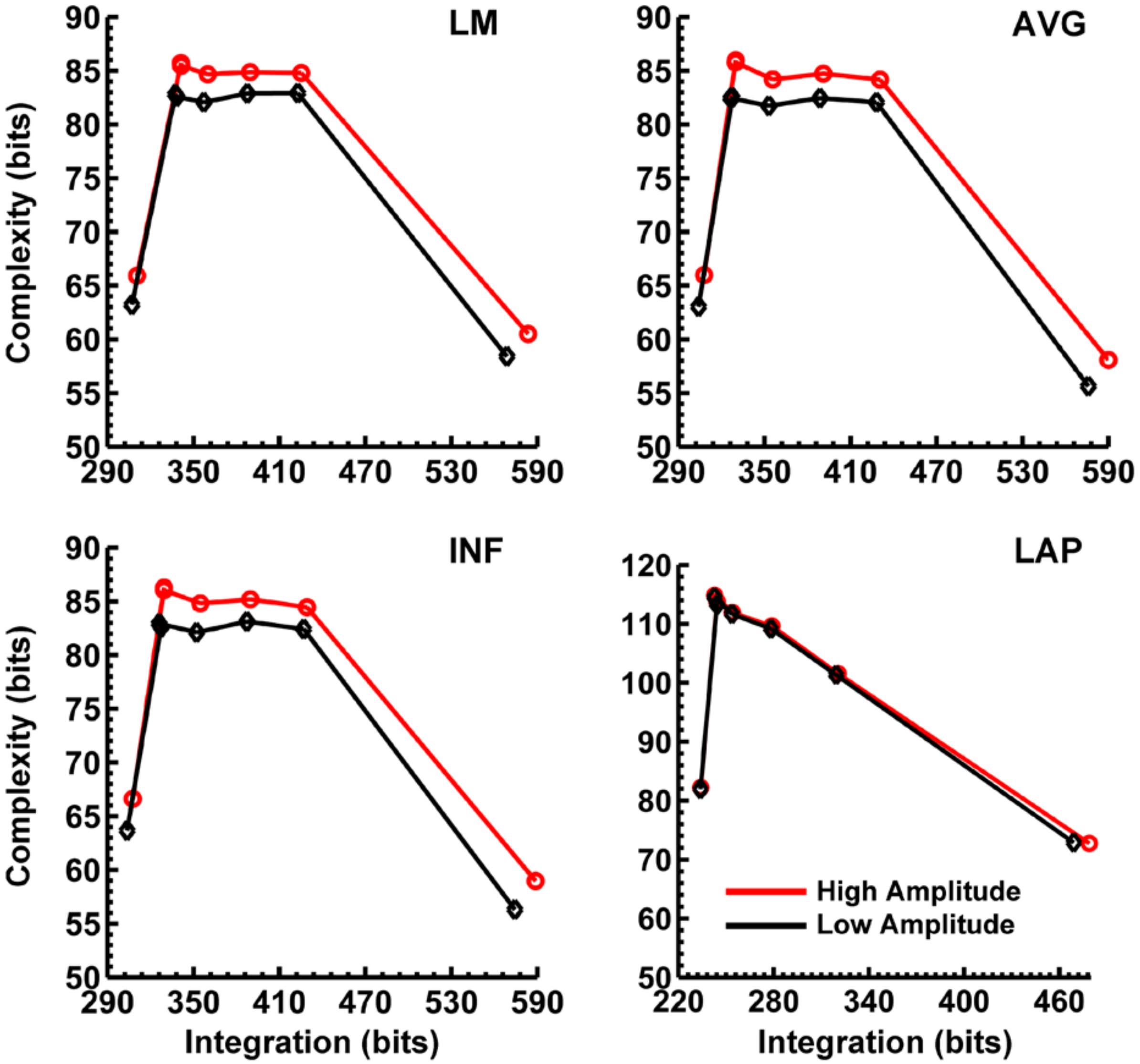

Figure S1. Interaction complexity $C_I(X)$ as a function of integration I(X) for theta-alpha-range simulated scalp EEG signal resulting from visual dipole source activity only. Data points are generated from the seven different interdipole dependency models and are ordered form left to right in terms of increasing dependency/integration (full independency model, independent model – level 2, independent model – level 1, interdependent model –level 3, interdependent model – level 2, interdependent model –level 1, full dependency model). Red lines = high amplitude simulations; black lines = low amplitude simulations. Data points reflect averages across twenty-two separate simulations; standard errors of simulated $C_I(X)$ and I(X) are too small to be displayed, but range from .03 - .27 bits.

Tables S3 and S4 compare source-level and scalp-level mean values for the theta-alpha band and beta band extrastriate sources, respectively. These tables illustrate two basic findings (see Results section of the main manuscript): 1) the Laplacian-referenced data yielded the highest complexity and lowest integration values of the four EEG references and 2) that the absolute complexity and integration values of the dipole sources are much lower than that observed at the scalp. This inflation is most likely due to volume conduction. These observations suggest that the LAP data yielded

Interaction Complexity $C_I(X)$											
**Source Dependency Level**	**Hi Amp Source**	**LMR**	**AVG**	**INF**	**LAP**		**Low Amp Source**	**LMR**	**AVG**	**INF**	**LAP**
Full Indep	1.66	65.92	65.98	66.62	82.22		1.62	63.22	63.04	63.66	81.97
Indep-2	48.84	85.74	85.99	86.28	113.81		48.05	82.81	82.52	82.59	113.26
Indep-1	57.82	85.46	85.78	86.04	114.83		56.68	82.59	82.45	82.94	114.52
Interdep-3	51.81	84.67	84.20	84.81	111.92		50.55	82.07	81.74	82.11	111.67
Interdep-2	39.77	84.88	84.74	85.19	109.63		38.73	82.90	82.46	83.12	109.16
Interdep-1	27.89	84.80	84.18	84.45	101.56		27.21	82.92	82.06	82.42	101.35
Full Dep	19.13	60.50	58.08	58.97	72.75		18.83	58.41	55.63	56.31	72.87
**Integration $I_I(X)$**											
**Source Dependency Level**	**Hi Amp Source**	**LMR**	**AVG**	**INF**	**LAP**		**Low Amp Source**	**LMR**	**AVG**	**INF**	**LAP**
Full Indep	1.98	310.57	307.81	307.61	233.99		1.93	307.01	303.94	303.67	233.83
Indep-2	51.74	341.27	329.87	329.48	244.33		50.96	337.33	327.47	327.41	244.24
Indep-1	61.89	341.45	329.69	329.42	242.76		60.54	338.35	326.36	326.08	242.66
Interdep-3	68.92	359.94	355.71	354.76	253.94		67.02	357.24	353.03	352.20	253.78
Interdep-2	83.20	389.55	390.98	389.68	278.79		80.30	387.60	388.69	387.28	278.28
Interdep-1	107.76	425.25	430.65	429.25	320.55		103.22	422.82	428.13	426.95	319.32
Full Dep	154.27	583.59	590.91	588.85	478.85		146.64	568.77	575.96	574.35	469.05

Table S3. Mean theta-alpha range extrastriate-only dipole simulation EEG source and scalp EEG interaction complexity $C_I(X)$ and integration I (X) by EEG reference and simulation amplitude. All values are in bits. Dep = Dependent; Interdep = Interdependent; Indep = Independent. Standard errors of simulated $C_I(X)$ and I(X) ranged from .01 - .10 bits for dipole sources and .04 - .15 bits for scalp-level measures.

the closest approximation to the true absolute dipole source integration values, but the worst approximation to the true absolute source complexity values. However, if one is interested in between-source dependency level differences across different EEG reference and experimental conditions (a situation of most interest to experimental psychologists, psychophysiologists, and cognitive neuroscientists), then a better criterion for EEG reference performance is the gradient of complexity or integration change across source dependency levels. Table S5 shows 1st-order dipole source-level and scalp-level EEG complexity and integration gradient changes between each source dependency level after averaging across high and low amplitude conditions. Importantly, the table also shows the root mean squared (RMS) error between the source-level and scalp-level complexity and integration gradients. The RMS error is computed across all six gradient points for each EEG reference. Although the LM reference yielded the lowest RMS gradient error for theta-alpha-range integration, the Laplacian-transformation yielded the lowest RMS gradient error for theta-alpha- and beta-range complexity and beta-range integration.

**Interaction Complexity $C_I(X)$**											
**Source Dependency Level**	**Hi Amp Source**	**LMR**	**AVG**	**INF**	**LAP**		**Low Amp Source**	**LMR**	**AVG**	**INF**	**LAP**
Full Indep	1.64	65.92	65.98	66.62	82.22		1.65	63.22	63.04	63.66	81.97
Indep-2	36.88	78.08	78.94	79.17	100.97		35.01	74.87	75.90	75.89	100.49
Indep-1	40.32	78.16	79.11	79.17	100.84		38.40	75.37	75.68	75.94	100.56
Interdep-3	34.75	78.89	79.96	80.66	98.85		33.45	76.11	77.16	77.73	98.65
Interdep-2	27.05	81.91	81.80	82.20	96.72		26.39	79.71	79.64	80.18	96.54
Interdep-1	25.60	82.00	81.67	81.95	91.60		25.22	79.78	79.53	79.64	91.56
Full Dep	24.82	63.29	60.89	61.71	76.28		24.57	61.33	58.54	59.27	76.59
**Integration $I_I(X)$**											
**Source Dependency Level**	**Hi Amp Source**	**LMR**	**AVG**	**INF**	**LAP**		**Low Amp Source**	**LMR**	**AVG**	**INF**	**LAP**
Full Indep	1.94	310.57	307.81	307.61	233.99		1.96	307.01	303.94	303.67	233.83
Indep-2	28.01	332.74	323.71	323.64	252.61		27.21	329.37	319.27	318.78	251.66
Indep-1	30.86	333.10	324.00	323.78	252.73		29.71	329.84	320.23	320.20	252.27
Interdep-3	41.63	350.05	346.65	345.35	261.35		40.03	347.22	343.77	342.60	260.97
Interdep-2	59.80	371.76	371.41	370.38	275.57		57.32	369.86	369.17	368.36	274.95
Interdep-1	85.76	401.27	404.52	403.24	305.16		82.05	399.32	401.97	401.19	303.89
Full Dep	127.98	543.22	549.67	547.71	439.67		122.08	528.93	535.66	533.92	430.86

Table S4. Mean beta range extrastriate-only dipole simulation EEG source and scalp EEG interaction complexity $C_I(X)$ and integration I (X) by EEG reference and simulation amplitude. All values are in bits. Dep = Dependent; Interdep = Interdependent; Indep = Independent. Standard errors of simulated $C_I(X)$ and I(X) ranged from .01 - .21 bits for dipole sources and .04 - .40 bits for scalp-level measures.

**Interaction Complexity $C_I(X)$**											
**Source Dependency Level Difference**	**Theta-Alpha Source**	**LMR**	**AVG**	**INF**	**LAP**		**Beta Source**	**LMR**	**AVG**	**INF**	**LAP**
Indep-2 –Full Indep	46.81	19.70	19.74	19.29	31.44		34.30	11.90	12.91	12.38	18.64
Indep-1 – Indep-2	8.80	-0.25	-0.14	0.05	1.14		3.41	0.29	-0.03	0.03	-0.03
Interdep-3 – Indep-1	-6.06	-0.66	-1.15	-1.03	-2.88		-5.26	0.73	1.16	1.64	-1.95
Interdep-2 – Interdep-3	-11.93	0.52	0.63	0.70	-2.40		-7.38	3.31	2.16	1.99	-2.12
Interdep-1 – Interdep-2	-11.70	-0.03	-0.48	-0.72	-7.94		-1.31	0.08	-0.12	-0.39	-5.05
Full Dep – Interdep-1	-8.57	-24.40	-26.26	-25.80	-28.64		-0.71	-18.58	-20.88	-20.31	-15.15
Across-Gradient RMS Error	–	15.21	15.45	15.47	11.64		–	12.80	12.97	12.99	9.29
**Integration $I_I(X)$**											
**Source Dependency Level Difference**	**Theta-Alpha Source**	**LMR**	**AVG**	**INF**	**LAP**		**Beta Source**	**LMR**	**AVG**	**INF**	**LAP**
Indep-2 –Full Indep	49.40	30.51	22.80	22.81	10.38		25.66	22.27	15.62	15.57	18.23
Indep-1 – Indep-2	9.87	0.60	-0.65	-0.70	-1.58		2.68	0.42	0.63	0.78	0.37
Interdep-3 – Indep-1	6.75	18.69	26.35	25.73	11.15		10.55	17.17	23.10	21.99	8.66
Interdep-2 – Interdep-3	13.79	29.99	35.47	35.00	24.68		17.73	22.18	25.08	25.40	14.10
Interdep-1 – Interdep-2	23.74	35.46	39.55	39.62	41.40		25.34	29.49	32.96	32.85	29.27
Full Dep – Interdep-1	44.97	152.15	154.05	153.50	154.02		41.13	135.78	139.42	138.60	130.74
Across-Gradient RMS Error	–	45.59	47.99	47.72	48.30		–	38.85	40.90	40.52	36.79

Table S5. Extrastriate-only dipole simulation complexity $C_I(X)$ and integration I (X) source dependency level gradients by EEG reference and frequency range. All values are in bits. Dep = Dependent; Interdep = Interdependent; Indep = Independent. RMS = Root Mean Squared.

References

Holm, S. (1979). A simple sequentially rejective multiple test procedure. *Scandinavian Journal of Statistics*, *6,* 65–70.

Lachaux, J.-P., Rodriguez, E., Le Van Quyen, M., Lutz, A., Martinerie, J., & Varela, F. J. (2000). Studying single-trials of phase synchronous activity in the brain. *Int. J. Bifurcat. Chaos, 10*, 2429–2439.

Massey, F. J. (1951). The Kolmogorov-Smirnov test for goodness of fit. *Journal of the American Statistical Association, 46*, 68–78.
